# Accurate real-time trajectory generation of circular motion using FIR interpolation: a trochoidal milling case study

**DOI:** 10.1007/s00170-025-15385-2

**Published:** 2025-04-09

**Authors:** David Wilkinson, Burak Sencer, Rob Ward

**Affiliations:** 1 Department of Automatic Control and Systems Engineering, University of Sheffield, Sheffield, S1 3JD UK; 2 Advanced Manufacturing Research Centre, University of Sheffield, Rotherham, S60 5TZ UK; 3 Mechanical Engineering Department, Oregon State University, Corvallis, OR USA

**Keywords:** Motion control, Circular interpolation, FIR filtering, Vibration suppression, Trochoidal milling

## Abstract

Subtractive manufacturing is undergoing a transformative shift towards sustainability and zero-defect manufacturing. This shift is driving the need for more efficient machining strategies such as dynamic milling. The real-time implementation of dynamic milling toolpaths, composed of circular and cycloidal curve patterns, is challenging due to the kinematic constraints in computer numerically controlled machine tools. Resulting from a rigorous analytical analysis of kinematics, the limitations of current approaches to finite impulse response (FIR) interpolation of circular arc (G02/G03) motion are addressed. A novel hybrid FIR interpolation method is presented which modifies the interpolation style depending on the fundamental geometry of commanded circular motion. The method globally satisfies kinematic constraints and tool centre point position tolerances during circular motion and allows consideration of machine dynamics (i.e., resonant frequencies) within the interpolation strategy. The proposed method outperformed current state-of-the-art methods during benchmarking tests which included a high-performance machine tool and two commercial controllers. Reductions of up to 38% in manufacturing cycle times were demonstrated when interpolating high-speed trochoidal toolpaths with the proposed method.

## Introduction

Subtractive manufacturing, and in particular high-speed machining (HSM), is undergoing a paradigm shift, with a focus on sustainability and zero-defect manufacturing. With this conceptual shift, machining toolpaths are becoming more complex in nature with an increased industrial uptake in using dynamic milling strategies such as trochoidal milling [1], in which circular and elliptical toolpath patterns are being used over traditional rectilinear motion. The dynamic toolpaths offer a range of advantages such as increasing material removal rates [2] and extending tool life [3].

**Fig. 1:**
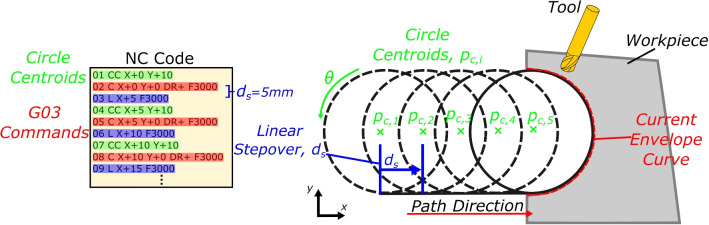
Quasi-trochoidal toolpath consisting of circular segments connected with a linear stepover, with corresponding NC code in HEIDENHAIN code format

These modern toolpath geometries lead to challenges in numerical controller (NC) interpolation, which is the generation of smooth position reference signals for the machine tool control system. To generate these signals, computer-aided manufacturing (CAM) software first outputs the complex toolpaths in G-code (ISO-6983) either as highly discretised point-to-point linear G01 commands or circular G02/G03 commands [4] followed by interpolation by the NC kernels (or interpolators). The interpolators compute these two types of commands very differently, and the resulting interpolation and its effect on machine tool response when using G02/G03 commands vs. G01 commands to program tool motion are not widely understood in academia or industry.

Trochoidal toolpath patterns are often generated in the CAM stage as quasi-trochoids with a linear stepover, rather than a cycloid curve [5]. Quasi-trochoidal curves can simply be approximated as a series of circles that are connected by a linear motion with a fixed stepover distance, as illustrated in Fig. 1. Whilst these approaches help design trochoidal toolpath patterns in the CAD/CAM stage, there is little consideration of the implementation of these toolpaths, thus ignoring the effects of NC interpolation on the accuracy of the machine tool motion along the trochoidal path.

CNC interpolation is a mature research field with two main approaches. The first is the traditional spline-based [6] and parametric curve [7] approach. These methods fit piecewise polynomials to the discrete cutter locations (CL) commanded in the part program (G-code) to create the smooth tool centre point (TCP) position signals. The most common are B-splines [8, 9] and NURBS [10–12], and solving these is computationally expensive—especially for large part programs [13]. The second, more recently addressed method is the filtering approach to linear interpolation using finite impulse response (FIR) filters [14, 15]. The filtering approach overcomes the computational burden of spline-based methods and is easily real-time implementable, due to FIR interpolation requiring a single step to generate the toolpath [14]—unlike spline-based and parametric curve methods, meaning that both the TCP position and feedrate signals are generated simultaneously [15]. Numerical controller manufacturers are now shifting to and adopting FIR technology [16, 17].

Another advantage of FIR filters is the capability to explicit control the frequency spectrum of the generated signals. Previous interpolation methods relied on general jerk-limited acceleration profiles to mitigate unwanted vibrations [6], whereas utilising FIR filters allows direct tuning of the frequency spectra of interpolated toolpaths [14], thus avoiding ill-effects of structural vibrations on the part such as increased surface roughness leading to part non-conformance.

Toolpath trajectory generation can be broken down into two main smoothing types: local and global corner smoothing, with the former being applied when CL points are sufficiently distanced from one another such that the first does not impact the second kinematically [14, 15, 18]. Global smoothing, on the other hand, is applied in the opposite situation wherein one tool motion command does directly impact another kinematically, with cornering error arising due to the overlapping of prior and posterior motion commands. The discretisation of circular toolpaths into small-segmented G01 commands warrants the use of global smoothing approaches. Such discretisations occur in the CAM stage during the generation of trochoidal toolpaths. With global smoothing, lookahead functions are often required to allow the NC interpolator to consider upcoming toolpath segments and adjust the feedrate to meet TCP tolerance. Multiple methods exist for such lookahead, including spline curve-based methods [19] and sliding mode control-inspired methods [20], with the latter allowing the incorporation of dynamic constraints within the toolpath smoothing stage.

Only a few seminal papers have addressed the global smoothing of G01 commands using FIR interpolation, with results showing that FIR interpolation of short-segment motion warrants a reduction in feedrate to control interpolation error during the global corner [21, 22]. The research by Tajima and Sencer constrained interpolation error only at the corners between linear segments and not along globally smoothed G01 paths, and thus cannot be applied to circular toolpaths discretised as G01 commands which require constraining of both path deviations and cornering error. Instead of discretising trochoidal paths into short linear segments, one can utilise longer-segment G02 arc paths and counteract the ill-effects of FIR interpolated short-segment motion.

Tajima et al. addressed FIR filter-based interpolation of G01 and G02/G03 commands [14] and developed a novel FIR interpolation method that considered TCP position and tool orientation tolerances whilst satisfying machine tool kinematic constraints. A key finding of this research is the frequency-dependent effects of filtering circular toolpath trajectories. As the FIR filter acts as a low-pass filter, the sinusoidal signals (i.e. the velocity profiles of the axis feed drives during circular paths) undergo both phase shift and attenuation, with the attenuation of the axial velocity profiles introducing interpolation error when moving through the circular motion. To circumvent this issue, the authors proposed a feedrate override factor $\alpha$ that reduces the commanded G02/G03 feedrate *F* to ensure that user-defined TCP position tolerance constraints are satisfied. Similarly, feedrate modulation using blending pulses has been used to control the contour error (or blending error) of G01 to G01 transitions in local smoothing [15], and therefore, such an approach is clearly viable for accurate interpolation of circular motion, however reducing feedrate *increases* the cycle time. The trade-off between toolpath accuracy and speed has been heavily researched and has formal international standards governing the standardisation of accuracy and interpolation of both linear and circular motion (see ISO 10791-6:2014 [23] and ISO 230-4:2022 [24]). Otsuki et al. proposed a two-dimensional method for evaluating the speed-accuracy trade-off in linear and rotary-axis systems [25], demonstrating the necessity for toolpath smoothing (AAI/ABI) and feedrate reduction in circular paths to meet tolerance requirements.

To avoid the necessity of reducing commanded feedrate during circular motion, Huang et al. [26] applied FIR filtering to circular toolpaths in a parametric space, named as parametric acceleration/deceleration for interpolation (PADI). The proposed algorithm first mapped circular motion onto a rotational coordinate frame, rather than performing interpolation in the Cartesian frame (referred to in their paper as acceleration/deceleration after interpolation (ADAI)). Applying FIR filters in a parametric mapping of the original toolpath completely removes the steady-state error introduced by the low-pass characteristics of the FIR filter [14]. The results of [26] showed that the proposed PADI algorithm resulted in cycle time reductions compared with the Cartesian axial-level FIR filtering method introduced by Tajima et al. [14], along with zero contour error. However, the optimal selection of the smoothing method based on the toolpath geometry was not wholly discussed, and there was no consideration of the frequency-domain effects of the resultant toolpath motion, opening up the possibility for excitation of machine resonant modes.

Ishizaki and Shamoto proposed a method of radius scaling to mitigate the error induced through FIR interpolation of circular arcs [27], in which the authors analysed the magnitude and phase changes caused by smoothing of circle motions of different angular frequencies. This method was successful in removing the error induced by the FIR filter; however, there was no consideration of kinematic constraints (acceleration and jerk) through the application of this method. Li et al. [7] proposed a method of FIR-filtered circular arc motions in the Cartesian coordinate system. Whilst their proposed methods are successful in allowing error-controlled blending of consecutive linear and arc segments, there still exists the need for a means of analytically defining and constraining axial and tangential acceleration and jerk during circular motions.

Traditionally, quasi-trochoidal toolpaths have been generated using discretised motion for both the linear and circular sections of the toolpath [28]. In the research by Rauch et al., the circular trochoidal path was discretised into 0.1 mm segments. Whilst this is clearly feasible for real-time implementation, the effects of global FIR interpolation of such short-segment toolpaths may lead to exceeding error tolerances [22]. More recently, research has been conducted in applying polynomial curves to generated trochoidal toolpaths [29], which allow the generation of both 3-axis and 5-axis circular trochoidal toolpaths through highly discretised linear G01 motion. For non-circular trochoidal paths, the use of NURBS curves has proven to be successful [30]; however, this requires the incorporation of nonlinear optimisation techniques during smoothing of the toolpath and thus may not be suitable for real-time implementation. This method also discretised the trochoidal motion into short segments before performing interpolation and smoothing.

In summary, there is a clear gap in research literature investigating the efficacy of utilising G02/G03 commands for the generation of trochoidal toolpaths. Overcoming this, this paper presents a detailed analysis of smoothed G02/G03-interpolated trochoidal motion, specifically tackling the issues surrounding kinematics-constrained generation of circular toolpath trajectories.

The authors present the following contributions: (1) a complete derivation of FIR-smoothed circular motion with filtering being applied in the Cartesian axial directions, (2) a novel kinematics-constrained zero interpolation error path-level FIR interpolation method that considers machine dynamics, and (3) a hybrid axial/path-level FIR method for time-optimised kinematics-constrained G02/G03 interpolation.

The proposed hybrid method uses only a single FIR filter to generate smooth error-controlled circular motion thereby simplifying the method for real-time on-the-fly interpolation. The frequency content of the signal can be adaptively controlled throughout the whole toolpath, and the method does not require feedrate reduction thereby demonstrating a reduction in cycle times compared to previous methods.

**Fig. 2:**
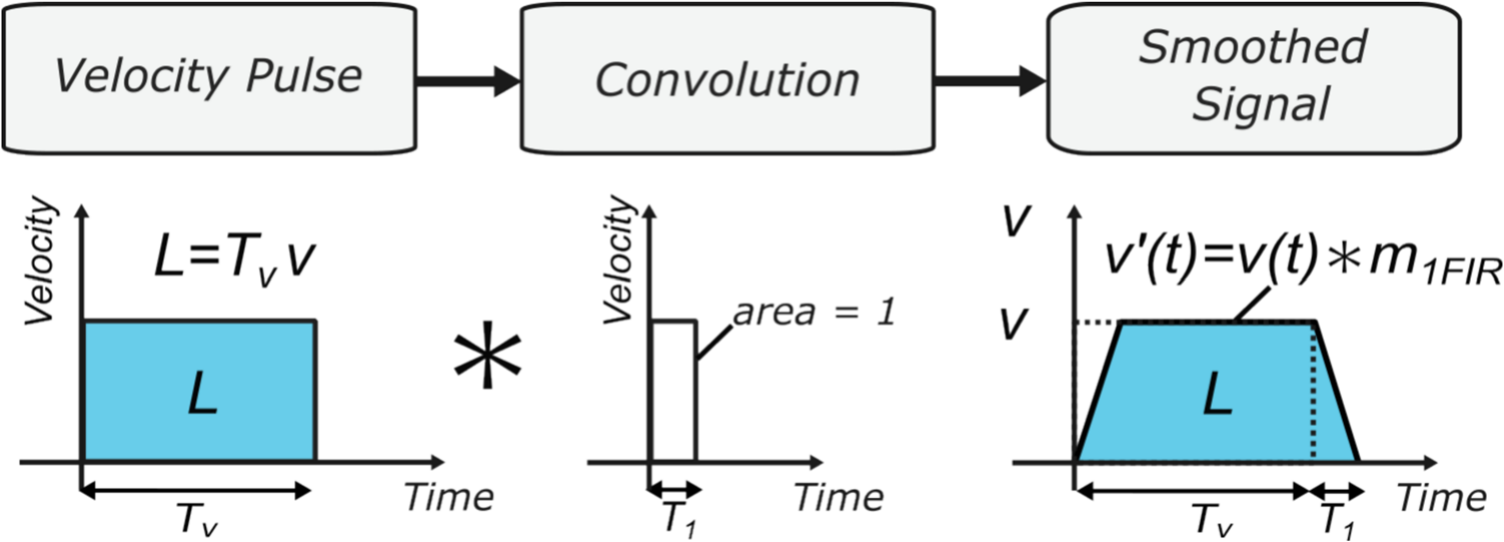
Signal smoothing via FIR filtering

The paper is presented as follows: first, the authors present a brief introduction to FIR filtering, followed by addressing the shortcomings in both methods of FIR interpolation at the Cartesian axial level. An analysis of path-level FIR filtering is then performed, in which low pass FIR filtering is applied in the feed direction, discussing the benefits of such an approach in eliminating interpolation error.

The Cartesian axial-level and path-level FIR interpolation methods are then combined to form a hybrid FIR interpolation method that modifies the interpolation style depending on the fundamental geometric limits of the motion. Finally, the proposed method is then benchmarked against a high-performance machine tool with a commercial controller and validated through experimental testing on a 5-axis machine tool.

## Circular interpolation within axis drive limits

This section will introduce the FIR interpolation of circular motion and address the kinematic constraints during circular motion. First, a short refresher of linear interpolation of G01 motion commands is presented, before deriving the analytical forms for ADAI FIR filter-based smoothing of circular motion in the Cartesian coordinate system.

### Real-time interpolation using FIR low pass filtering

Previous research introduced methods of linear interpolation using FIR filtering [14, 15, 18]. Therefore, only a short précis of FIR filtering-based linear interpolation is included here for completeness.

The transfer function of a first-order FIR filter is defined in the Laplace domain as follows:

1 $$\begin{aligned} M_i(s)=\frac{1}{T_i} \frac{1-e^{-s T_i}}{s}, i=1 \ldots N \end{aligned}$$

where *s* is a complex number, and $T_{i}$ is the time constant of the $i^{th}$ filter. The area under the curve is maintained at unity due to the filter gain being set to $1/T_i$, and therefore, no area scaling occurs when convolving the filter with a signal. In the frequency domain, the FIR filter $M_i\left( j \omega \right)$ is represented by the following:

2 $$\begin{aligned} M_i(j \omega )=\frac{1}{T_i} \frac{1-e^{-j \omega T_i}}{j \omega }, \end{aligned}$$

and the time domain representation is given by the following:

3 $$\begin{aligned} m_i(t)&=\mathcal {L}^{-1}\left\{ M_i(s)\right\} =\frac{u(t)-u\left( t-T_i\right) }{T_i} \end{aligned}$$

4 $$\begin{aligned} u&= {\left\{ \begin{array}{ll}1, &  t \ge 0 \\ 0, &  t<0\end{array}\right. } \end{aligned}$$

where $m_i(t)$ the impulse response of the FIR filter and $\mathcal {L}$ is the Laplace operator.

Convolution between a velocity pulse *v*(*t*) and $m_i(t)$ (in either the time or frequency domain) will increase the order of the convolved signal (as shown in Fig. 2) and results in a smoother interpolated signal,

5 $$\begin{aligned} v^{\prime }(t)=v(t) * m_i(t), \end{aligned}$$

where $v^{\prime }(t)$ represents the interpolated velocity signal. Further convolution with FIR filters increases the order of the signal and is used to generate higher-order interpolated motion control trajectories for NC operations. The next section will show the interpolation method applied to G01 commands.

### Linear interpolation of point-to-point motion

The start and end positions of a linear G01 command in 3 axes can be represented by $\textbf{P}_{\textrm{s}}$ and $\textbf{P}_{\textrm{e}}$, respectively, with $\textbf{P} =\left[ P_{x}, P_{y}, P_{z}\right] ^{T}$ being the TCP positions in Cartesian coordinates. The tool displacement *L* is calculated from the Euclidean norm of the vector between the two commanded positions, $L=\left\| \textbf{P}_{e}-\textbf{P}_{s}\right\| _{2}$. The velocity pulses of each axis $\left( v_{x}, v_{y}, v_{z}\right)$ are calculated by multiplying the feed pulse *v*(*t*) by the unit velocity vector $\textbf{u}=(\mathbf {P_{e}}-\mathbf {P_{s}}) /\Vert \mathbf {P_{e}}-\mathbf {P_{s}}\Vert _{2}$:

6 $$\begin{aligned} \frac{d \textbf{P}(\textbf{t})}{d t}=\dot{\textbf{P}}(\textbf{t})=v(t) \textbf{u}=\begin{bmatrix} v_{x}(t) \\ v_{y}(t) \\ v_{z}(t) \end{bmatrix}, \end{aligned}$$

where $$\dot{\textbf{P}}(\textbf{t})$$ represents the time derivative of the linear displacement. Convolving the impulse response of the FIR filter with the axis velocity pulses $$(v_{x}$$, $$v_{y}$$, $$v_{z})$$ generates interpolated axis velocity profiles:

7 $$\begin{aligned} \frac{d \textbf{P}^{\prime }(t)}{d t}=\dot{\textbf{P}}^{\prime }(t)=\begin{bmatrix} {v_x}^{\prime }(t) \\ {v_y}^{\prime }(t) \\ {v_z}^{\prime }(t) \end{bmatrix}=\dot{\textbf{P}}(t) * m_i(t),\end{aligned}$$

where ($v^{\prime }_{x}$,$v^{\prime }_{y}$,$v^{\prime }_{z}$) with the prime notation represent the filtered (and thereby smoothed) axis velocity commands. Integrating the filtered axis velocity commands yields the interpolated position commands:

8 $$\begin{aligned} \textbf{P}^{\prime }(t)=\begin{bmatrix} {s_{x}}^{\prime }(t) \\ {s_{y}}^{\prime }(t) \\ {s_{z}}^{\prime }(t) \end{bmatrix}=\int _{0}^{t} \begin{bmatrix} {v_x}^{\prime }(\tau ) \\ {v_y}^{\prime }(\tau ) \\ {v_z}^{\prime }(\tau ) \end{bmatrix} \, d \tau + \textbf{P}_s. \ \end{aligned}$$

where ${s_i}^\prime (t)$ represents the smoothed axial displacement signal of the $i^{\text {th}}$ axis, and $\textbf{P}_s$ represents the initial starting point of the motion. The kinematic profiles (velocity, acceleration, and jerk) can be derived analytically by evaluating the convolution integral between the velocity pulse and the rectangular impulse response of the FIR filter. The maximum acceleration $A_{\max }$ and jerk $J_{\max }$ are derived from their analytical forms. In the case of using 2 identical FIR filters for smoothing, the FIR time constant $T_{1}$ can be selected based on the closed-form analytical smoothed motion profiles [15] to meet the kinematic constraints as follows:

9 $$\begin{aligned} T_{1} =\text {max}\left\{ \frac{\Delta F }{A_{\max }},\sqrt{\frac{\Delta F}{J_{\max }}}\right\} . \end{aligned}$$

The following section will show selection of the linear motion (G01) time constant does not universally hold across all types of toolpaths and may lead to exceeding machine kinematic limits and TCP position tolerances *especially* in circular and trochoidal toolpaths.

### Real-time interpolation of circular motion

In NC operations, circular motion can be commanded either from multiple G01 commands forming a highly discretised circle which is generated and defined entirely in the CAM stage, or directly as G02/G03 commands. This section will focus on FIR interpolation for G02/G03 commands.

**Fig. 3:**
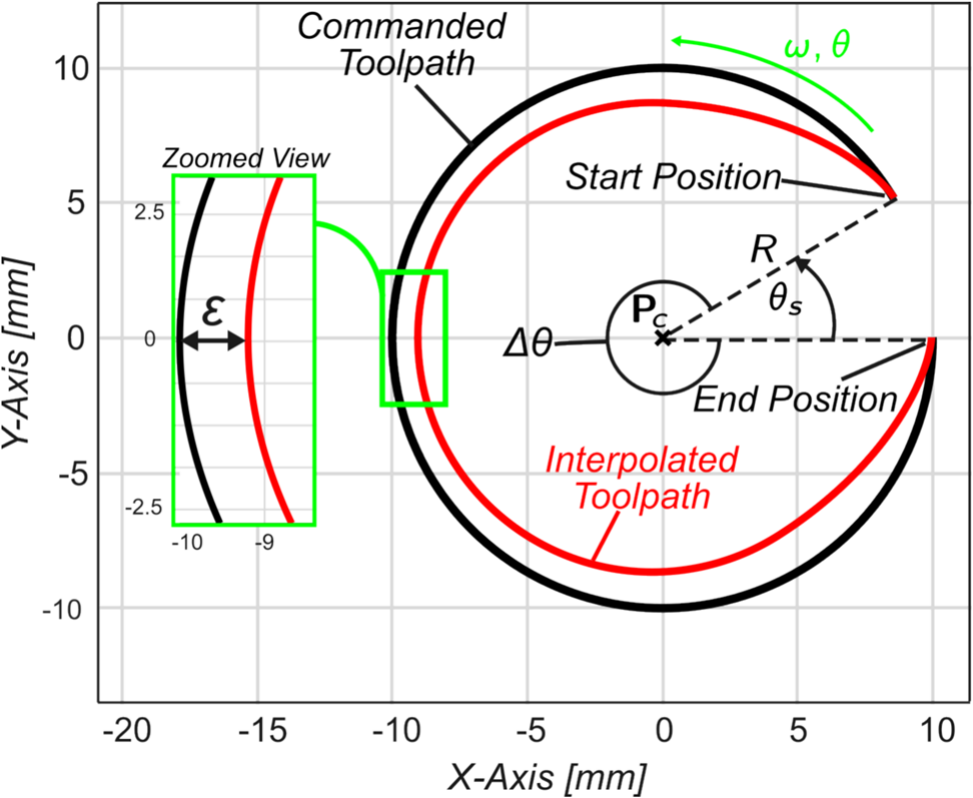
Smoothed G03 circular motion, with non-zero start angle

During circular motion, the displacement *L* the tool travels around the arc of the circular path is calculated as $L = R\Delta \theta$ where *R* is the arc radius defined by the G02/G03 command and $\Delta \theta = \theta _{e} - \theta _{s}$ is the angular displacement between start and end commanded angular positions, $\theta _{s}$ and $\theta _{e}$ respectively. The feed pulse *F* is broken down in Cartesian axial velocity components $v_x(t)$ and $v_y(t)$, and the circular motion commands are generated at the rotational (circular) frequency $\omega _c$, where $\omega _c = F/R$ as follows:

10a $$\begin{aligned} s_x(t)&=x_c + R \cos \left( \omega _c t+\theta _s\right) , \end{aligned}$$

10b $$\begin{aligned} s_y(t)&=y_c + R \sin \left( \omega _c t+\theta _s\right) , \end{aligned}$$

where $s_i$ represents the displacement profile of the $i^{th}$ axis of motion where $i\in (x,y)$, and $\textbf{P}_{\textrm{c}} = \left[ x_{c}, y_{c}\right] ^{T}$ represent the arc centre coordinates seen in Fig. 3.

An important distinction must be made here between linear and circular motion when interpolating using FIR filters. The time constants $T_{i}$ in linear motion are selected based upon their analytically defined acceleration and jerk profiles using Eq. 9. However, the kinematic behaviour of circular motion is fundamentally different from linear motion. In linear motion, only the tangential velocity, tangential acceleration, and/or tangential jerk are required to calculate the time constant that satisfies the kinematic constraints. In circular motion, as will be shown in the following section, both tangential and normal, and thereby centripetal kinematics, must be taken into consideration.

**Fig. 4:**
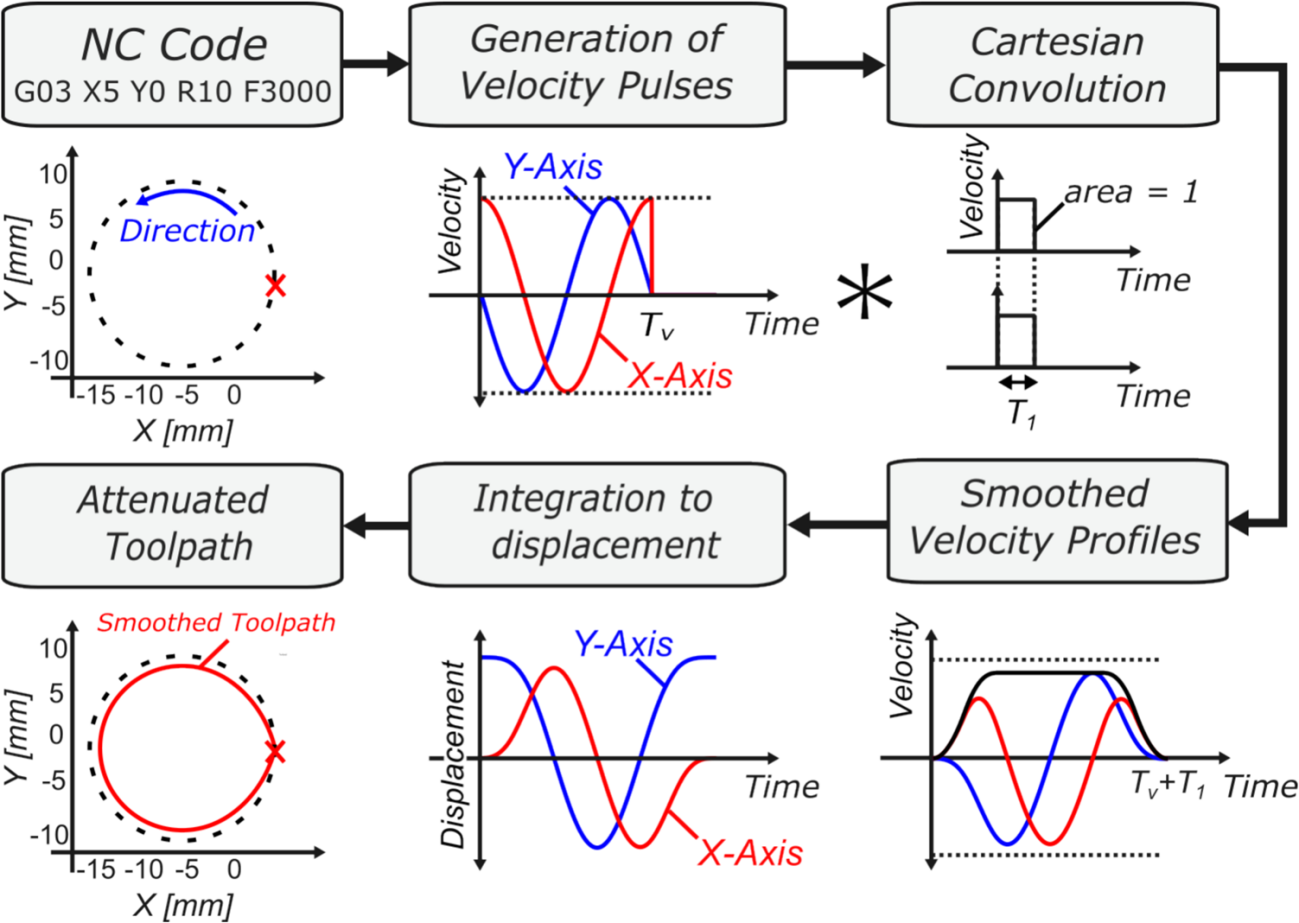
Cartesian-FIR interpolation of single G03 command

### Kinematics of circular interpolation with FIR filtering in Cartesian axes

This section will analytically evaluate smoothing of G02/G03 commands where each Cartesian axis is individually filtered and address the TCP position error and kinematic constraints. The current state of the art in FIR interpolation focuses on smoothing velocity pulses through convolution of axial feed pulses with two to three cascading FIR filters [18]. A minimum of two FIR filters is required to allow the generation of jerk-limited acceleration profiles (JLAP) in the smoothing of linear G01 motions, which leads to velocity signals of $C^2$ parametric continuity. However, in circular motion, the derivatives of motion (displacement, velocity, acceleration, jerk, snap, and so forth) are continuous and thus have $C^\infty$ continuity, and as such, the requirement of utilising at minimum two FIR filters is negated. Therefore, in this proposed method, a single FIR filter (1FIR) will be used for deriving motion profiles (Fig. 4).

To begin, the unfiltered axial displacement profiles (i.e. the commanded toolpath) can be expressed as

11a $$\begin{aligned} s_x(t)&= R \cos \theta (t), \end{aligned}$$

11b $$\begin{aligned} s_y(t)&= R\sin \theta (t). \end{aligned}$$

**Fig. 5:**
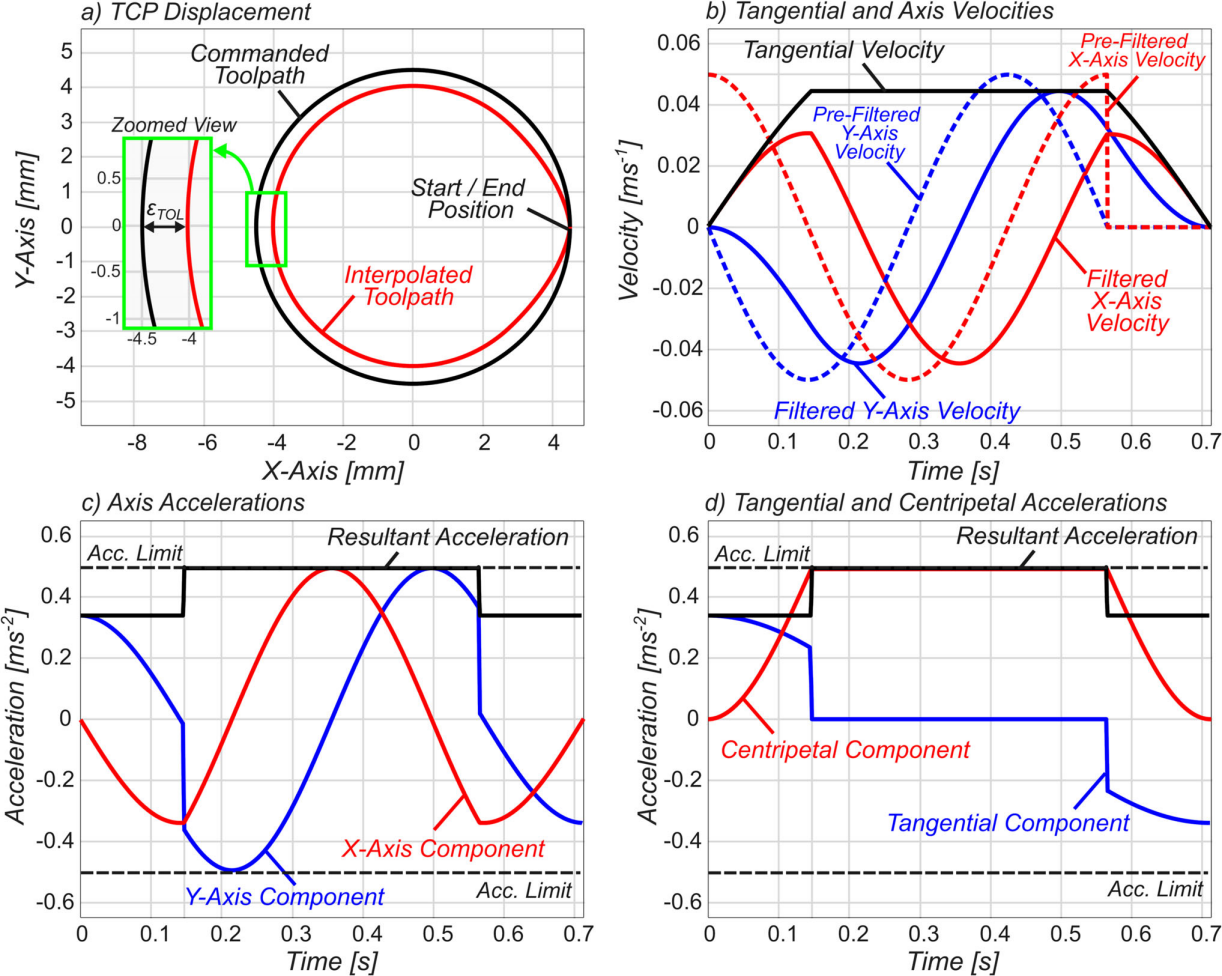
Cartesian axial-level FIR interpolated motion

Taking the derivative of the displacement profiles with respect to time leads to the axial velocity profiles (as shown by the dotted lines in Fig. 5b):

12a $$\begin{aligned} v_x(t)&= -\omega _c R \sin \theta (t), \end{aligned}$$

12b $$\begin{aligned} v_y(t)&= \omega _c R\cos \theta (t), \end{aligned}$$

where $\omega _c = \dot{\theta }(t) = F/R$ is the constant angular frequency of the circle (circular frequency) that is dependent on commanded feedrate *F* and radius *R* of the G02/G03 command. This is equivalent to decomposing the feed pulse *v*(*t*) into axial components prior to filtering. To aid in simplifying the analysis, one can represent the angle $\theta (t)$ as a function of the circular frequency $\omega _c$:

13 $$\begin{aligned} \theta (t) = \int ^t \omega _c \ dt = \omega _c t + \theta _s, \end{aligned}$$

where $\theta _s = \theta (t)$ at $t=0$, defined as the initial angle at the beginning of the circular motion thereby allowing representation of the axial velocity profiles in Eq. 12a as

14a $$\begin{aligned} v_x(t)&= -\omega _c R \sin \left( \omega _c t + \theta _s\right) , \end{aligned}$$

14b $$\begin{aligned} v_y(t)&= \omega _c R \cos \left( \omega _c t + \theta _s\right) . \end{aligned}$$

To obtain the smoothed Cartesian axial velocity profiles, Eqs. 14a and 14b are each convolved with the single FIR filter, ${v_i}^\prime (t) = {v_i}(t) * m_{1FIR}(t)$ where $i\in (x,y)$, resulting in the smoothed velocity profiles ${v_x}^\prime (t)$ and ${v_y}^\prime (t)$ as defined in Eqs. 15 and 16, respectively. Equations 15 and 16 are important. From these equations, the smoothed axis displacement profiles can be derived along with the acceleration and jerk profiles. Integrating Eqs. (15) and (16) and adding the initial $\theta _s$-dependent conditions results in the smoothed axis displacement profiles ${s_x}^\prime (t)$ and ${s_y}^\prime (t)$, which can be found at Eqs. A1 and A2, respectively. The remainder of the derivation will focus on the *x*-axis profiles. The reader may wish to verify that the same relationships hold when filtering is applied on the Cartesian *y*-axis kinematic profiles, noting that the derivation is universally applicable on any planar circular motion in a three-axis system, with the modification of the initial angle $\theta _s$.

15 $$\begin{aligned} {v_x}^\prime (t)&= \left\{ \begin{array}{ll} \frac{R}{T_1} \cos \left( \omega _c t + \theta _s \right) - \frac{R}{T_1} \cos \left( \theta _s\right) &  0 \le t< T_1, \\ \frac{2R}{ T_1}\sin \left( \frac{\omega _c T_1}{2}\right) \cos \left( \omega _c t + \phi \right) &  T_1 \le t< T_v, \\ \begin{aligned} &  \frac{R}{T_1}\cos \left( \omega _c T_v +\theta _s\right) - \frac{R}{T_1}\cos \left( \omega _c (t - T_1) + \theta _s \right) \end{aligned} &  T_v \le t < T_v + T_1, \\ 0 &  T_v + T_1 \le t, \end{array} \right. \end{aligned}$$

16 $$\begin{aligned} {v_y}^\prime (t)&= \left\{ \begin{array}{ll} \frac{R}{T_1} \sin \left( \omega _c t + \theta _s \right) - \frac{R}{T_1} \sin \left( \theta _s\right) &  0 \le t< T_1, \\ \frac{2R}{ T_1}\sin \left( \frac{\omega _c T_1}{2}\right) \sin \left( \omega _c t + \phi \right) &  T_1 \le t< T_v, \\ \begin{aligned} &  \frac{R}{T_1} \sin \left( \omega _c T_v +\theta _s\right) - \frac{R}{T_1} \sin \left( \omega _c (t - T_1) + \theta _s \right) \end{aligned} &  T_v \le t < T_v + T_1, \\ 0 &  T_v + T_1 \le t, \end{array} \right. \end{aligned}$$

$$\begin{aligned} \text {where} \quad \phi&= \theta _s + \text {atan2}\big (\sin \left( \omega _c T_1 \right) , 1 - \cos \left( \omega _c T_1 \right) \big ). \end{aligned}$$

It must be observed that, whilst the unfiltered *x*-axis and *y*-axis displacement profiles take the form $\cos (f(t))$ and $\sin (f(t))$, respectively, the smoothed profiles contain time-varying sinusoidal terms of form $t\sin (f(t))$, which adds complexity to analysing and constraining TCP error due to the functions not being periodic.

Differentiating Eqs. (15) and (16) permits the maximum axial acceleration to be calculated which includes the additional centripetal terms. In particular, the analysis highlights the toolpath design parameters that lead to breaching of the machine tool kinematic limits. The axial acceleration profile, $A_x(t)$, can be seen in Eq. 18. Subsequent differentiation of the axial acceleration profile Eq. (18) leads to the interpolated jerk profile $J_x(t)$ of Eq. 19. It must be noted that, depending on the value of $\omega _c T_1$ and initial condition $\theta _s$, the maximum acceleration and jerk for each axis may fall in either the first $(0 \le t < T_1,)$, second $(T_1 \le t < T_v)$, or third $(T_v \le t < T_v + T_1)$ piecewise profile. Therefore, the derivation of maximum jerk and acceleration during circular motion must also be performed in a piecewise manner. Taking the maximum of each piecewise function and expressing $\omega _c$ as *F*/*R*, the maximum instantaneous axial acceleration and jerk can be expressed as follows:

17a $$\begin{aligned} A_{x,y,\max }&= \max \left\{ \frac{F}{T_1}, \frac{2F}{ T_1}\sin \left( \frac{\omega _c T_1}{2}\right) \right\} , \end{aligned}$$

17b $$\begin{aligned} J_{x,y,\max }&= \max \left\{ \frac{{F}^2 }{T_1R}, \frac{2F^2}{ T_1 R}\sin \left( \frac{\omega _c T_1}{2}\right) \right\} . \end{aligned}$$

In the case that $\big |\sin (\omega _c T_1/2)\big | > 1/2$, axial acceleration and jerk will reach a maximum during the steady-state period of circular motion, in which $t \in [T_1, T_v)$, whereas if $\big |\sin (\omega _c T_1/2)\big | < 1/2$, the maximum acceleration and jerk will occur in the filter acceleration/deceleration period.

18 $$\begin{aligned} {A_x}^\prime (t)&= \left\{ \begin{array}{ll} - \frac{\omega _c R}{T_1} \sin \left( \omega _c t + \theta _s \right) &  0 \le t< T_1, \\ -\frac{2\omega _c R}{ T_1}\sin \left( \frac{\omega _c T_1}{2}\right) \sin \left( \omega _c t + \phi \right) &  T_1 \le t< T_v, \\ \frac{\omega _c R}{T_1}\sin \left( \omega _c (t - T_1) + \theta _s \right) &  T_v \le t < T_v + T_1, \\ 0 &  T_v + T_1 \le t, \end{array} \right. \nonumber \\ \end{aligned}$$

19 $$\begin{aligned} {J_x}^\prime (t)&= \left\{ \begin{array}{ll} - \frac{{\omega _c}^2 R}{T_1} \cos \left( \omega _c t + \theta _s \right) &  0 \le t< T_1, \\ -\frac{2{\omega _c}^2 R}{ T_1}\sin \left( \frac{\omega _c T_1}{2}\right) \cos \left( \omega _c t + \phi \right) &  T_1 \le t< T_v, \\ \frac{{\omega _c}^2 R}{T_1}\cos \left( \omega _c (t - T_1) + \theta _s \right) &  T_v \le t < T_v + T_1, \\ 0 &  T_v + T_1 \le t, \end{array} \right. \end{aligned}$$

To summarise, this section demonstrated that choosing the FIR filter time constant $T_1$ based on FIR interpolation of linear G01 commands (i.e., solely considering acceleration between $0\le t < T_1$) may *breach* acceleration limits during the steady-state period of circular motion. Hence, designing $T_1$ using the analytical method for G01 interpolation (as in Eq. 9) does not universally satisfy the kinematic constraints across all toolpath conditions. The next section addresses this and proposes a method to select $T_1$ which satisfies all kinematic constraints.

#### FIR filter parameter selection with respect to drive limits

It has been demonstrated (see Eq. 17) that maximum axial and tangential acceleration is a function of commanded feedrate *F* and the circular radius *R*. Maximum acceleration can either occur in the transient (filter acceleration/deceleration) period or in steady-state circular motion, dependent on the value of $\sin \left( \omega _c T_1/2\right)$. Therefore, to ensure maximum acceleration is within constraints in both the transient and steady-state period, the FIR filter time constant $T_1$ must be constrained for both conditions,

20 $$\begin{aligned} \underbrace{\frac{F}{T_1}}_{\textit{transient}} \le A_{\max } \cap \underbrace{\frac{2F}{ T_1}\sin \left( \frac{\omega _c T_1}{2}\right) }_{\textit{steady-state}}\le A_{\max }. \end{aligned}$$

This constraint is nonlinearly dependent on $$T_1$$. Employing a Taylor series expansion of $$\sin (\omega _c T_1/2)$$ (up to the cubic term), substituting $$\omega _c = F/R$$, and rearranging for $$T_1$$, the constraint on $$T_1$$ due to acceleration limits (i.e., $$T_{1,acc})$$ can be expressed as

21 $$\begin{aligned} T_{1,Acc} \ge \max \left\{ \frac{F}{A_{\max }}, \frac{2\sqrt{6} R}{F^2} \sqrt{ F^2 - {A_{\max }} R} \right\} . \end{aligned}$$

It must be noted that the second constraint only has a real solution if $F^2/R \ge A_{\max }$, i.e., if the *centripetal* acceleration does not exceed the acceleration limits. If $A_{\max } > F^2/R$, then all positive $T_1$ values hold, resulting in the following decision of $T_1$ for acceleration,

22 $$\begin{aligned} T_{1,Acc} \ge \left\{ \begin{array}{ll} \max \left\{ \frac{F}{A_{\max }}, \alpha \right\} &  \textit{if } A_{\max } < \frac{F^2}{R}, \\ \frac{F}{A_{\max }} &  \textit{otherwise}. \end{array} \right. \end{aligned}$$

where $\alpha = \frac{2\sqrt{6} R}{F^2} \sqrt{ F^2 - {A_{\max }} R}$. Similarly, linearising Eq. 17 using Taylor series expansion leads to the following jerk-limited ($T_{1, \text { jerk}}$) constraint for choosing the FIR time constant,

23 $$\begin{aligned} T_{1,Jerk} \ge \left\{ \begin{array}{ll} \max \left\{ {\frac{F^2}{J_{\max } R}}, \beta \right\} &  \textit{if } J_{\max } < \frac{F^3}{R^2}, \\ {\frac{F^2}{J_{\max } R}} &  \textit{otherwise}, \end{array} \right. \end{aligned}$$

where $\beta = \frac{2\sqrt{6} R}{F^2} \sqrt{({F^3 - {J_{\max }} R^2})/{F} }$. The $T_1$ selection is therefore conditional on whether maximum allowable jerk $J_{\max }$ is within the centripetal jerk $F^3/R^2$. It must be noted that the Taylor series approximations used are all approximations of the function $\sin \left( \omega _c T_1/2\right)$ and serve as suitable approximations when $\omega _c T_1 < 2$.

Thus far, the analysis has addressed the kinematic constraints. The following section will propose a method to control the TCP position error.

#### Interpolation error control

This section will analyse Cartesian-FIR-filtered circular motion in terms of resultant TCP position error, and it can be defined as the Euclidean distance between the smoothed circular profile and the commanded profile [14]. As the entire ideal toolpath motion is a circle with radius *R* and centroid $(x_c,y_c)$, the ideal tangential TCP motion can be defined as the magnitude of the axial motion profiles [14], and hence, the TCP error can be defined as

24 $$\begin{aligned} \varepsilon _{TCP}(t) = R - \sqrt{{s_x}^\prime (t)^2 +{s_y}^\prime (t)^2}, \end{aligned}$$

where ${s_x}^\prime (t)$ and ${s_y}^\prime (t)$ are the smoothed time-varying axial displacement profiles described in Eqs. A1 and A2. Substituting the profiles during the steady-state period, where error is maximum, leads to

25 $$\begin{aligned} \varepsilon _{\max } = R - \frac{2R}{{\omega _c} T_1}\sin \left( \frac{\omega _c T_1}{2}\right) . \end{aligned}$$

Using the Taylor series expansion of $\sin (\omega _cT_1/2)$ up to the cubic term allows the approximation of maximum error as

26 $$\begin{aligned} \varepsilon _{\max } \approx \frac{F^2 {T_1}^2}{24R}. \end{aligned}$$

This maximum error $\varepsilon _{\max }$ reached during the steady-state period must be within the user-defined TCP position error tolerance $\varepsilon _{Tol}$,

27 $$\begin{aligned} \frac{F^2 {T_1}^2}{24R} \le \varepsilon _{Tol}. \end{aligned}$$

Using Eq. 27, the FIR filter time constant $T_1$ can be tuned to attain the desired TCP tolerance $\varepsilon _{Tol}$. Using the Taylor series expansion of $\sin (\omega _cT_1/2)$ up to the cubic term and rearranging Eq. 27 for $T_1$ results in

28 $$\begin{aligned} T_1 \le \frac{2\sqrt{6}}{F} \sqrt{\varepsilon _{Tol} R}. \end{aligned}$$

By tuning $T_1$ in Eq. 28, one guarantees that the interpolated profile will not exceed user-defined TCP position tolerances.

The final piece of the puzzle is to address the structural modes of the machine tool. Therefore, in addition to the acceleration, jerk, and TCP position tolerance constraints, the frequency content of generated trajectories must also be taken into consideration to ensure no excitement of structural dynamic modes occurs. The FIR filter can be tuned to dampen the resonant frequencies of the motion system by selection of $T_{1}$ [14] through

29 $$\begin{aligned} T_1 = \frac{2\pi }{\omega _r} \ge \frac{1}{f_r}, \end{aligned}$$

where $\omega _r$ is the frequency of the first structural dynamic mode of the machine tool in rad/s, and $f_r$ is the frequency in hertz. At this value of $T_1$, maximum attenuation of the signal occurs; however, setting this condition as a *lower bound* for the FIR filter time constant allows avoidance of exciting dynamic modes, albeit not total attenuation at the resonant frequency $f_r$. Therefore, the conditions that determine the feasibility of the toolpath generation can be derived through evaluating the overall inequality

30 $$\begin{aligned} \max \left\{ \frac{1}{f_r}, T_{1,Acc}, T_{1,Jerk} \right\} \le T_1 \le \underbrace{\frac{2\sqrt{6}}{F} \sqrt{\varepsilon _{Tol} R}}_\textit{TCP tolerance}, \end{aligned}$$

where $T_{1,Acc}$ and $T_{1,Jerk}$ are chosen from Eqs. 22 and 23, respectively.

If inequality Eq. 30 does not hold, then Cartesian axial-level FIR interpolation *cannot* be applied for generating error-constrained circular motion, and one must either resort to changing the toolpath in the CAM stage, or utilising the *path-level FIR* interpolation method introduced in the next section. Based on inequality Eq. 30, one can also observe that setting the FIR time constant based on solely the linear motion design criteria, as in [14] is not sufficient for ensuring that the resultant circular motion is both error-constrained *and* acceleration/jerk-constrained. In the case that the inequality does not hold, the manufacturing engineer must redesign the toolpath through either altering the feedrate *F* or increasing the G02/G03 command circle radius *R* in the CAM stage.

It should be noted that there is also an upper bound on the precision of generated toolpaths when utilising Cartesian axial-level FIR interpolation. Based on the inequality Eq. 30, one can derive a limit $\varepsilon _{LIM}$ on TCP tolerance that is still feasible through the Cartesian axial-level FIR filtering method,

31 $$\begin{aligned} \varepsilon _{LIM} = \frac{F^2 {T_{1,\max }}^2}{24 R}, \end{aligned}$$

where $T_{1,\max } = \max \left\{ \frac{1}{f_r}, T_{1,Acc}, T_{1,Jerk} \right\}$. For example, with a modest feedrate of 3000 mm/min, a circle radius of 10 mm, a resonant frequency of 10 Hz, and maximum acceleration and jerk of 2 ms$^{-2}$ and 10 ms$^{-3}$, respectively, the minimum achievable TCP tolerance is calculated as 104.2 $\mu$m.

#### Illustrative example

Figure 5 shows the results of Cartesian axial-level FIR filtering of circular motion where radius $R=4.5$ mm, $F=3000$ mm/min, $\varepsilon _{Tol}=500\, \mu$m and $A_{max}=0.5$ ms$^{-2}$. Selecting $T_{1}$ based on Eq. 30 ensures the TCP position tolerance and acceleration constraints are met as shown in Fig. 5 a, c and d respectively.

Figure 5a shows the non-zero steady-state interpolation error that arises due to applying FIR filtering in the Cartesian frame. This is further evidenced by the difference between the pre-filtered and filtered velocity profiles in Fig. 5b, which shows the amplitude attenuation and the phase shift induced through FIR filtering. The nonlinearity of the piecewise acceleration profile can be observed in Fig. 5c, which shows the difference in peak acceleration in the transient filter period ($t = 0$ to 0.15 s) and the steady-state circular motion. Parallels can be observed between the tangential and centripetal acceleration and the axial components of acceleration, showing that the fluctuations in the axial components are due to changes in both tangential and centripetal acceleration during the transient filter period.

This section has addressed the shortcomings in Cartesian axial-level FIR filtering of circular motions. However, to circumvent the frequency-domain attenuation one may instead, as will be proposed in the following section, apply path-level filtering on the tangential toolpath motion by prior transformation of the toolpath into the angular domain.

## Kinematics of circular interpolation with FIR filtering in the feed direction

As discussed in Section 2.4.2, performing circular interpolation in the Cartesian coordinate system results in a steady-state error (see Fig. 5d) that fluctuates around the circle arc, warranting either a reduction in feedrate or increase in FIR filter time constant—both of which increase total cycle time. However, transforming the problem from the Cartesian coordinate system into the rotational motion frame and applying FIR interpolation in the feed direction removes the filtering-induced error, thereby eliminating the need to reduce feedrate during circular motion.

**Fig. 6:**
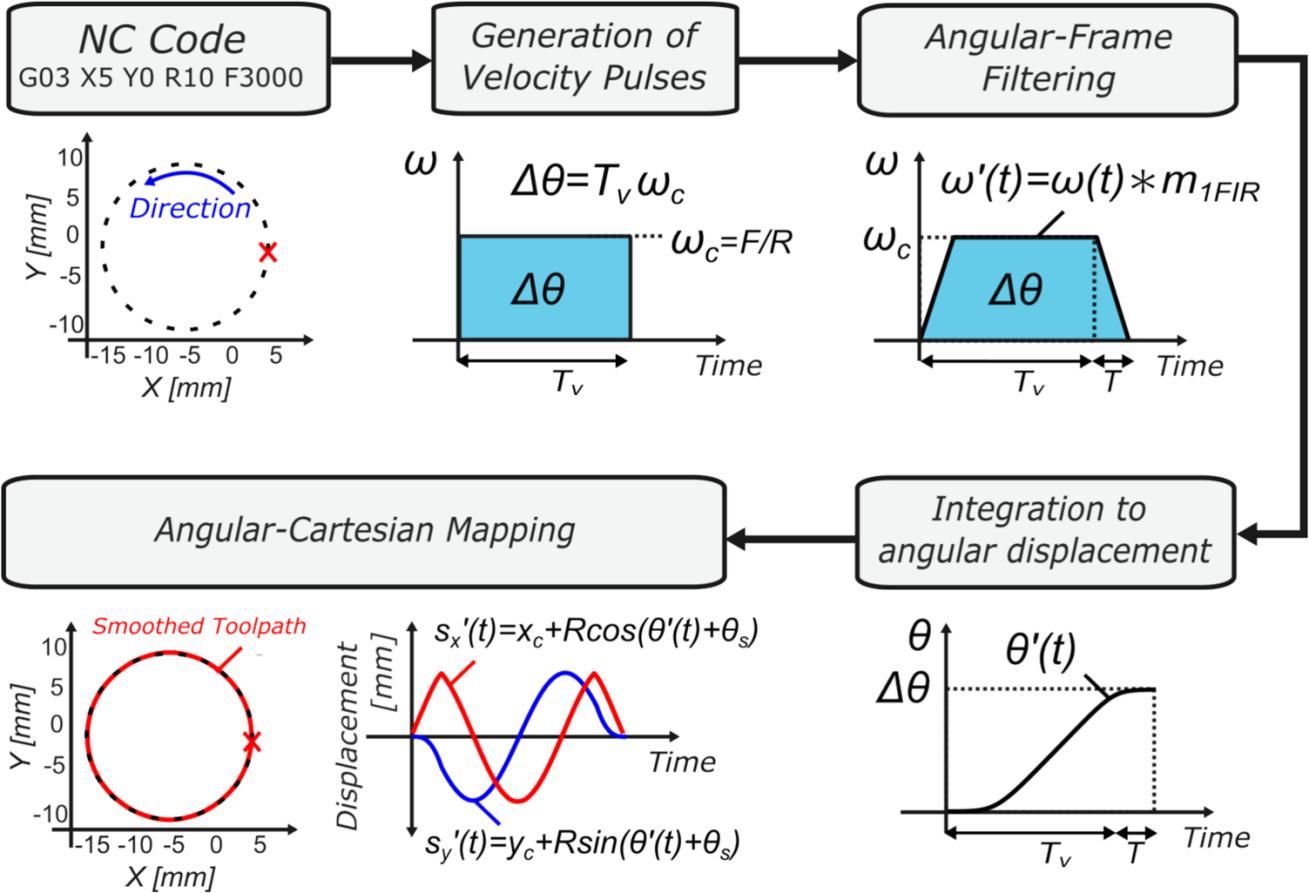
Path-level FIR filtering for circular motion

This section introduces a parametric mapping of circular interpolation that formulates the trajectory generation problem as a function of angular displacement and velocity along the circular arc G02/G03 command, which will from herein be referred to as path-level FIR. The analyses presented within this section will alternate between Cartesian and angular coordinate systems. The mappings between Cartesian axial displacements $s_x(t)$ and $s_y(t)$, and angular displacement $\theta (t)$, and between tangential velocity *v*(*t*) and angular velocity $\omega (t)$ are as follows:

32a $$\begin{aligned} s_x(t)&= x_c + R\cos \left( \omega (t) \, t\right) , \end{aligned}$$

32b $$\begin{aligned} s_y(t)&= y_c+ R \sin \left( \omega (t) \, t\right) , \end{aligned}$$

32c $$\begin{aligned} \omega (t)&= \dot{\theta }(t) = \frac{v(t)}{R}. \end{aligned}$$

In steady state, the angular velocity $\omega (t)$ is equal to the tangential velocity (i.e., commanded feedrate *F*) divided by the circle radius, $\omega _{c} = F/R$. FIR filter-based smoothing can then be performed on the angular velocity pulse $\omega (t)$ through convolution with the single FIR filter profile $m_{1FIR}(t)$ as in Eq. 4,

33 $$\begin{aligned} \omega '(t) = \omega (t) * m_{1FIR}(t), \end{aligned}$$

where $\omega (t)$ is the angular velocity pulse, defined as

34 $$\begin{aligned} \omega (t) = \left\{ \begin{array}{ll} \omega _c &  0\le t < T_v, \\ 0 &  T_v \le t. \end{array} \right. \end{aligned}$$

Evaluating the convolution integral Eq. 33 results in the smoothed angular velocity profile $\omega '(t)$,

35 $$\begin{aligned} {\omega ^{\prime }}(t)= \left\{ \begin{array}{ll} \frac{\omega _c}{{T_1}} t &  0 \le t<T_1 , \\ \omega _c &  T_1 \le t<T_v, \\ -\frac{\omega _c}{{T_1}}\tau (t) &  T_v \le t < T_v+T_1 , \\ 0 &  T_v+T_1 \le t, \end{array} \right. \end{aligned}$$

where $$\tau (t) = t - \left( T_v + T_1\right) $$.

As seen in Fig. 6, the angular velocity pulse increases in duration by $T_d$, where here $T_d = T_1$. Due to the FIR filter having a unity area, the total area under the curve (being the angular displacement $\Delta \theta$) remains unchanged, with the displacement in the full circular rotation being $\Delta \theta = 2\pi$ in the clockwise direction for a complete circular G02 motion. The smoothed angular displacement $\theta ^\prime (t)$ is piecewise defined by integrating the smoothed angular velocity profile $\omega ^\prime (t)$ of Eq. 35,

36 $$\begin{aligned} {\begin{matrix} {\theta ^{\prime }}(t)& = \int ^t \omega ^\prime (t) \ dt \\   & = \left\{ \begin{array}{ll} \frac{\omega _c}{2{T_1}} t^2 &  0 \le t<T_1, \\ \omega _c t - \frac{\omega _c T_1}{2} &  T_1 \le t<T_v , \\ -\frac{\omega _c}{2{T_1}}\tau (t) ^2 + \omega _c T_v &  T_v \le t < T_v+T_1, \\ \omega _c T_v &  T_v+T_1 \le t. \end{array} \right. \end{matrix}} \end{aligned}$$

State-of-the-art approaches to FIR filter-based toolpath smoothing would, at this point, require an additional FIR filter to result in a $C^2$-continuous velocity profile to allow the constraining of higher derivatives of motion (acceleration and jerk) in line with machine tool kinematic limits. Instead, at this point, the remainder of the analysis will be presented in Cartesian form, thereby utilising the sinusoidal motion profiles of the *xy*-axes Eq. 10 thus providing the $C^{\infty }$ continuity required for constraining higher derivatives of motion.

To obtain smoothed Cartesian axial displacement profiles $${s_x}^\prime (t)$$ and $${s_y}^\prime (t)$$, the smoothed angular displacement profile $$\theta ^\prime (t)$$ can then be substituted into the Cartesian axial profiles $$s_x(t)$$ and $$ s_y(t)$$ as in Eq. 32,

37a $$\begin{aligned} {s_x}^\prime (t)&= x_c + R \cos \left( \theta ' (t) + \theta _s\right) , \end{aligned}$$

37b $$\begin{aligned} {s_y}^\prime (t)&= y_c + R \sin \left( \theta ' (t) + \theta _s\right) . \end{aligned}$$

The smoothed angular displacement ${\theta }^\prime (t)$ is piecewise-defined Eq. 36; therefore, it follows that the resulting Cartesian axial motion profiles are piecewise-defined, as seen in Eq. 38. Taking the time derivative of the smoothed *x*-axis displacement profile results in the smoothed *x*-axis velocity, seen in Eq. 39. To allow analysis and constraining of maximum axial acceleration and jerk, one can take the derivative and second derivative of Eq. 39, which leads to the axial acceleration profile ${A_x}^\prime (t)$ of Eq. 40 and jerk profile ${J_x}^\prime (t)$, which can be seen in Eq. 41. The analytical axial acceleration Eq. 40 and jerk profiles Eq. 41 will be used in the following section to ensure the constraints are satisfied during G02/G03 circular motion.

38 $$\begin{aligned} {s_x}^\prime (t)&= \left\{ \begin{array}{ll} x_c + R\cos \left( \frac{\omega _c}{2T_1} t^2+ \theta _s \right) &  0 \le t< T_1, \\ x_c + R\cos \left( \omega _c t - \frac{\omega _c T_1}{2}+ \theta _s \right) &  T_1 \le t< T_v, \\ x_c + R\cos \left( -\frac{\omega _c}{2T_1} \tau (t)^2 - \omega _c T_v + \theta _s \right) &  T_v \le t < T_v + T_1, \\ x_c + R\cos \left( \omega _c T_v+ \theta _s \right) &  T_v + T_1 \le t. \end{array} \right. \end{aligned}$$

39 $$\begin{aligned} {v_x}^\prime (t)&= \left\{ \begin{array}{ll} -\frac{\omega _c R}{T_1} t \sin \left( \frac{\omega _c}{2T_1} t^2+ \theta _s \right) &  0 \le t< T_1, \\ -\omega _c R \sin \left( \omega _c t - \frac{\omega _c T_1}{2}+ \theta _s \right) &  T_1 \le t< T_v, \\ \frac{\omega _c R}{T_1} \tau (t) \sin \left( -\frac{\omega _c}{2T_1} \tau (t)^2 - \omega _c T_v + \theta _s \right) &  T_v \le t < T_v + T_1 , \\ 0 &  T_v + T_1 \le t. \end{array} \right. \end{aligned}$$

40 $$\begin{aligned} {A_x}^\prime (t)&= \left\{ \begin{array}{ll} \begin{aligned} &  -\frac{\omega _c R}{T_1} \sin \left( {\theta _1}^\prime (t)+ \theta _s \right) - \frac{{\omega _c}^2 R}{{T_1}^2}t^2 \cos \left( {\theta _1}^\prime (t)+ \theta _s \right) \end{aligned} &  0 \le t< T_1, \\ - {\omega _c}^2 R\cos \left( {\theta _2}^\prime (t)+ \theta _s \right) &  T_1 \le t< T_v, \\ \begin{aligned} &  \frac{ \omega _c R}{T_1} \sin \left( {\theta _3}^\prime (t)+ \theta _s \right) - \frac{ {\omega _c}^2 R}{{T_1}^2}\tau (t)^2 \cos \left( {\theta _3}^\prime (t)+ \theta _s \right) \end{aligned}&  T_v \le t < T_v + T_1 , \\ 0 &  T_v + T_1 \le t, \end{array} \right. \end{aligned}$$

41 $$\begin{aligned} {J_x}^\prime (t)&= \left\{ \begin{array}{ll} \begin{aligned} &  - \frac{3 {\omega _c}^2 R}{{T_1}^2}t \cos \left( {\theta _1}^\prime (t)+ \theta _s \right) +\frac{{\omega _c}^3 R}{{T_1}^3}t^3 \sin \left( {\theta _1}^\prime (t)+ \theta _s \right) \end{aligned} &  0 \le t< T_1, \\ \ {\omega _c}^3 R\sin \left( {\theta _2}^\prime (t)+ \theta _s \right) &  T_1 \le t< T_v, \\ \begin{aligned} &  - \frac{3 {\omega _c}^2 R}{{T_1}^2}\tau (t) \cos \left( {\theta _3}^\prime (t) + \theta _s \right) - \frac{{\omega _c}^3 R}{{T_1}^3}\tau (t)^3 \sin \left( {\theta _3}^\prime (t) + \theta _s \right) \end{aligned} &  T_v \le t < T_v + T_1, \\ \ 0 &  T_v + T_1 \le t, \end{array} \right. \end{aligned}$$

$$\begin{aligned} \text {where} \quad {\theta _1}^\prime (t) = \frac{\omega _c}{2T_1}t^2, \quad&{\theta _2}^\prime (t) = \omega _c t - \frac{\omega _c T_1}{2}, \text {and } {\theta _3}^\prime (t) = -\frac{\omega _c}{2T_1} \tau (t)^2 - \omega _c T_v. \end{aligned}$$

**Fig. 7:**
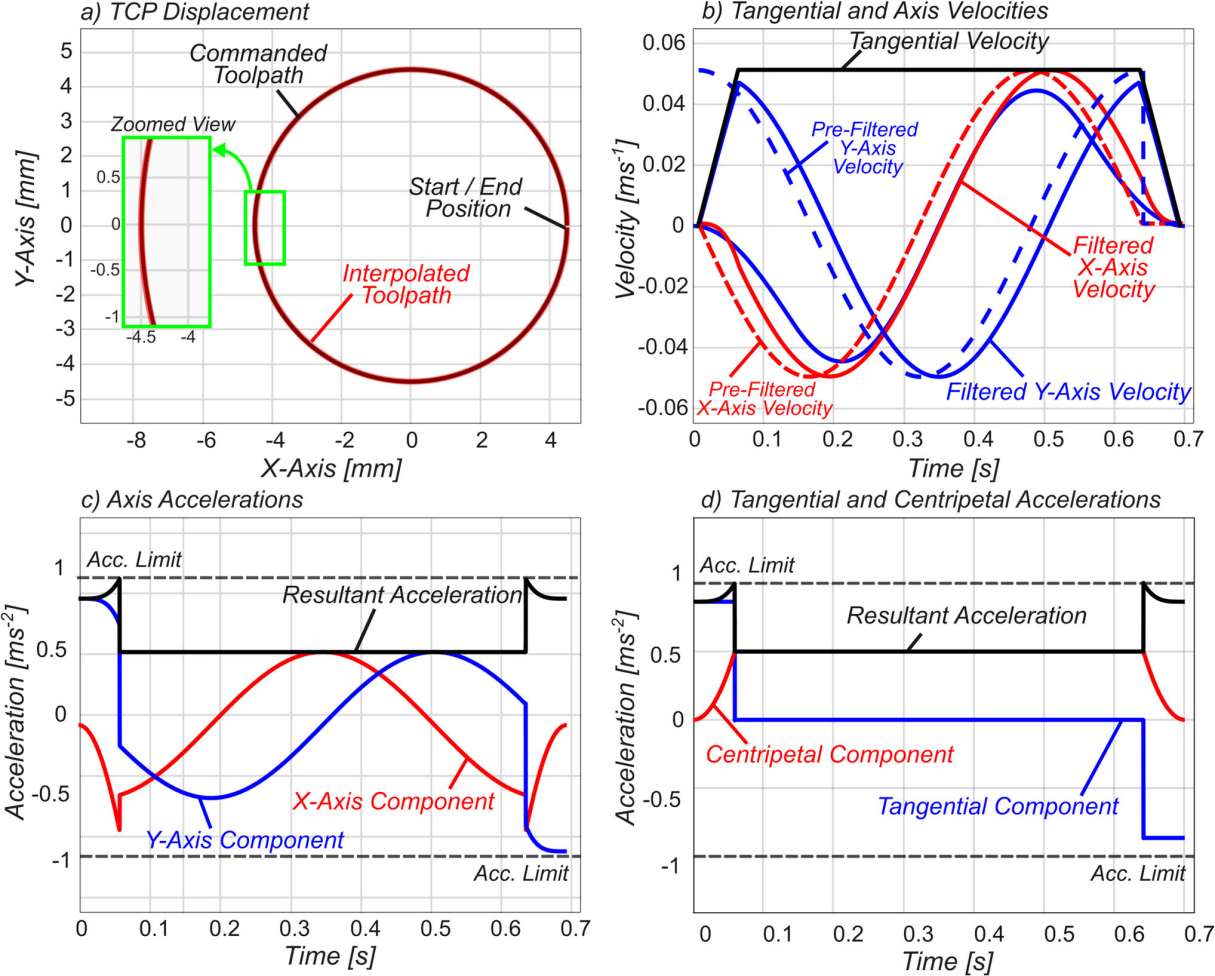
Path-level FIR interpolated motion

### Path-level FIR generation of acceleration and jerk-limited circular motion

CNC machine tool manufacturers set acceleration limits based on the physical limits of the feed drives. Therefore, generated toolpath motions *must* ensure that the *resultant* motion is within each axis’ kinematic constraints. As Eq. 43 shows, by constraining the resultant motion, one *automatically* constrains axial motion, where the resultant TCP acceleration $A_r$ is defined as

43 $$\begin{aligned} A_r = \sqrt{{A_x}^2 + {A_y}^2} = \sqrt{{A_t}^2 + {A_c}^2}, \end{aligned}$$

and is composed of both tangential acceleration $A_t$ and centripetal acceleration $A_c$.

The acceleration profiles have local maxima/minima in each of the piecewise functions. However, when considering axial kinematic limits, one must ensure that all local maxima/minima are within constraints, i.e., the global maximum and minimum points must be within the axial acceleration constraint

44 $$\begin{aligned} \max \left| {A_i}^\prime (t)\right| \le \max \left| {A_r}^\prime (t)\right| \le A_{\max }, \end{aligned}$$

where $i = x,y$. To verify this, one must first derive the maximum acceleration in each piecewise function of the smoothed Cartesian axial acceleration segments of Eq. 40. Substituting the smoothed axial acceleration Eq. 40 and the equivalent derived *y*-axis profile into Eq. 43 results in the following piecewise resultant acceleration profile:

45 $$\begin{aligned} {A_r}^\prime (t) = \left\{ \begin{array}{ll} \sqrt{\frac{ {\omega _c}^2 R^2 \left( {T_1}^2 + {\omega _c}^2 t^4 \right) }{{T_1}^4}} &  0 \le t< T_1, \\ {\omega _c}^2 R &  T_1 \le t< T_v, \\ \sqrt{\frac{ {\omega _c}^2 R^2 \left( {T_1}^2 + {\omega _c}^2 \tau (t)^4 \right) }{{T_1}^4}} & T_v \le t < T_v + T_1, \\ 0 &  T_v + T_1 \le t. \end{array} \right. \end{aligned}$$

The resultant acceleration is continuously increasing in the first segment as time progresses (due to the positive $t^4$ term—see Fig. 7c), and therefore, the maximum can be evaluated at the upper boundary of the first function’s time interval $t= T_1$. The second resultant acceleration profile is the steady-state circular motion portion, which has a magnitude independent of the path-level FIR filter time constant $T_1$ and can be seen to be constant across the time interval $T_1 \le t <T_v$.

The third piecewise resultant acceleration profile is a mirror image of the first piecewise resultant acceleration profile, mirrored about time $t = (T_v + T_1)/2$, and thus has the same absolute maximum value. Therefore, the maximum smoothed resultant acceleration can occur either in the transient (filter acceleration/deceleration period) or the steady-state circular motion. Upon evaluating the maximum function, one can observe that the first term is *always* larger than the second term (as both $\omega _c > 0$ and $T_1 > 0$):

46 $$\begin{aligned} {{A_r}^\prime }_{\max }&= \max \left\{ \underbrace{\frac{{\omega _c} R}{T_1}\sqrt{{\omega _c}^2 T_1^2 + 1}}_{\textit{transient}}, \underbrace{{\omega _c}^2 R}_{\textit{steady-state}} \right\} \end{aligned}$$

47 $$\begin{aligned} \Rightarrow {{A_r}^\prime }_{\max }&= \frac{{\omega _c} R}{T_1}\sqrt{{\omega _c}^2 T_1^2 + 1}. \end{aligned}$$

The resultant jerk ${J_r}^\prime$ is achieved in a similar manner, i.e., substituting the axial jerk Eq. 41 (and the equivalent y-axis derived profile) into the following:

48 $$\begin{aligned} J_r = \sqrt{{J_x}^2 + {J_y}^2} = \sqrt{{J_t}^2 + {J_c}^2} \end{aligned}$$

which yields the following piecewise resultant jerk:

49 $$\begin{aligned} {J_r}^\prime (t)\hspace{-1mm} = \hspace{-1mm} \left\{ \begin{array}{ll} \sqrt{\frac{ {\omega _c}^4 t^2 R^2 \left( 9{T_1}^2 + {\omega _c}^2 t^4 \right) }{{T_1}^6}} &  0 \le t< T_1, \\ {\omega _c}^3 R &  T_1 \le t< T_v, \\ \sqrt{\frac{ {\omega _c}^4R^2 \tau (t)^2 \left( 9{T_1}^2 + {\omega _c}^2 \tau (t)^4 \right) }{{T_1}^6}} & T_v \le t < T_v + T_1, \\ 0 &  T_v + T_1 \le t. \end{array} \right. \end{aligned}$$

From the smoothed resultant jerk profile Eq. 49, one can observe that, like the resultant acceleration Eq. 45, the first piecewise function is continuously increasing as time progresses (due to the positive $t^2$ and $t^4$ terms), and therefore, the maximum can also be evaluated at the upper boundary of the first function’s time interval $t= T_1$. One can observe that the maximum of the first piecewise function is *always* larger than the second function, and therefore, the maximum smoothed resultant jerk is as follows:

50 $$\begin{aligned} {{J_r}^\prime }_{\max }&= \max \left\{ \underbrace{\frac{{\omega _c}^2 R}{T_1}\sqrt{{\omega _c}^2 T_1^2 + 9}}_{\textit{transient}}, \underbrace{{\omega _c}^3 R}_{\textit{steady-state}} \right\} \end{aligned}$$

51 $$\begin{aligned} \Rightarrow {{J_r}^\prime }_{\max }&= \frac{{\omega _c}^2 R}{T_1}\sqrt{{\omega _c}^2 T_1^2 + 9}. \end{aligned}$$

Equation 50 showcases that if the maximum jerk is constrained in the transient period such that ${{J_r}^\prime (t)}_{\max } \le J_{\max }$, then the steady-state circular motion is *automatically* jerk-constrained, and within kinematic limits.

### Path-level FIR filter parameter selection with respect to drive limits

Based on the analysis in the previous section, the FIR filter time constant can now be calculated for the various constraint conditions. From the maximum resultant acceleration Eq. 46 and jerk Eq. 50, one can choose the FIR filter time constant $T_1$ to ensure that circular motion during the filter acceleration and deceleration periods is within kinematic constraints. Starting with the resultant acceleration Eq. 46 and solving the transient solution for $T_1$ leads to the following:

52 $$\begin{aligned} T_{1,Acc} \ge F R \sqrt{\frac{1}{{A_{\max }}^2 R^2 - {F}^4 }}. \end{aligned}$$

where the subscript *Acc* represents the $T_1$ value for satisfying acceleration constraints. This only has a valid solution when ${F^2}/{R} < A_{\max }$. Solving Eq. 50 for $T_1$ (i.e., $T_1 = T_{1,Jerk}$) results in the following:

53 $$\begin{aligned} T_{1,Jerk} \ge 3F^2 R \sqrt{\frac{1}{{J_{\max }}^2 R^4 - {F}^6 }}, \end{aligned}$$

which has a valid solution only if ${F^3}/{R^2} < J_{\max }$. FIR filters must also be tuned to satisfy frequency-domain constraints, that is, the FIR filter frequency $f_1 = 1/T_1$ should be tuned to attenuate any signals at the resonant frequency of the machine $f_r$, which can be achieved through ensuring that $f_1$ is lower than the machine’s first resonant mode $f_r$:

54 $$\begin{aligned} f_1 \le f_r \Rightarrow \frac{1}{f_r} \le T_1. \end{aligned}$$

The overall time constant $T_1$ can then be set to constrain acceleration, jerk, and resonate modes through taking the maximum of the acceleration, jerk, and resonant frequency $T_1$ criteria:

55 $$\begin{aligned} \frac{1}{f_r} \le {\max }\left\{ T_{1,Acc},T_{1,Jerk} \right\} \le T_1, \end{aligned}$$

resulting in the overall $T_1$ selection criteria in Eq. 56. As a guideline, CAM engineers should design toolpaths to ensure that G02/G03 commands are achievable given a desired machine’s kinematic limits—regardless of the interpolation method. Therefore, the following conditions for circular motion *should* be satisfied in the design stage:

$$\begin{aligned} \frac{F^2}{R} \le A_{\max } \cap \frac{F^3}{R^2} \le J_{\max }. \end{aligned}$$

This condition also applies to quasi-trochoidal toolpaths programmed as G02/G03 commands.

### Hybrid axial/path-level FIR interpolation

**Fig. 8:**
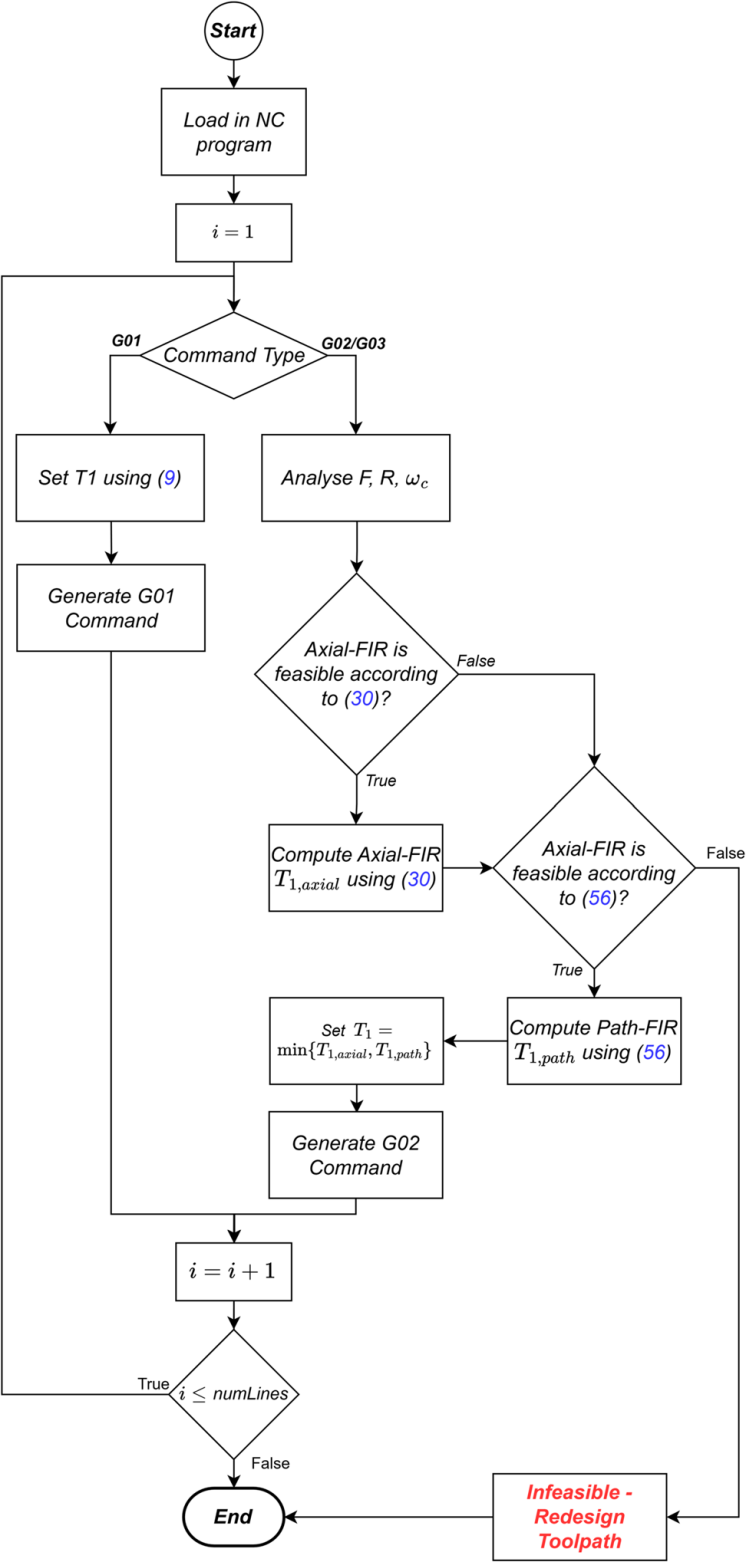
Proposed hybrid axial/path-level FIR interpolation method for G01 and G02/G03 commands

To summarise thus far, two novel methods for designing FIR filters for interpolation of circular motion have been presented. The two methods complement one another; the Cartesian axial-level FIR method allows interpolation of G02/G03 commands with centripetal acceleration/jerk larger than the respective kinematic limits, but has a limit in terms of achievable TCP error tolerance, whereas the path-level FIR method induces zero TCP error, but can only be used when centripetal acceleration and jerk are within permitted limits. Therefore, one can utilise both methods dependent on the circular command being interpolated, as depicted in the flow chart in Fig. 8.

This hybrid approach allows the application of FIR interpolation to a wider range of circular toolpaths; in cases where path-level FIR interpolation is infeasible (due to centripetal acceleration and/or jerk exceeding kinematic limits), the Cartesian axial-level FIR algorithm can be applied (Appendix).

56 $$\begin{aligned} \underbrace{ \frac{1}{f_r} }_\textit{resonance} \le \max \left\{ \underbrace{ F R \sqrt{\frac{1}{{A_{\max }}^2 R^2 - {F}^4 }}}_\textit{acceleration}, \underbrace{3F^2 R \sqrt{\frac{1}{{J_{\max }}^2 R^4 - {F}^6 }}}_\textit{jerk} \right\} \le T_1 . \end{aligned}$$

In most cases, where both axial-level and path-level FIR interpolation can be applied, one can calculate the shortest time constant $T_1 = \min \{T_{1, axial}, T_{1,path}\}$ and ensure faster overall cycle time whilst keeping within kinematic and geometric constraints.

The next section will experimentally validate the hybrid axial/path-level FIR method and benchmark the performance improvements with the proposed method against both the state-of-the-art methods in FIR interpolation (as introduced by [14]), as well as against high-performance 5-axis machining centres with integrated commercial-off-the-shelf CNC interpolator units.

## Experimental validation

This section will first benchmark the proposed hybrid Cartesian axial-level/path-level FIR filtering method against a leading commercial controller and then validate the proposed method through experimental trials. The proposed method will be compared against the conventional FIR filtering methods introduced by Tajima et al. [14], which will be referred to as the “linear-FIR” method. The linear-FIR approach uses two FIR filters for generating jerk-limited motion, with filter time constant $T_1$ selection based on Eq. 9. The key performance indicators that will be benchmarked are the cycle time and the satisfaction of constraints—TCP tolerance, acceleration, and jerk limits.

**Table 1:** TNC640 interpolator parameters for case study #1

Parameter	Value
NC interpolator sample frequency	1000 Hz
Maximum axial acceleration	3.1 ms$^{-2}$
Maximum axial jerk	157 ms$^{-3}$

**Fig. 9:**
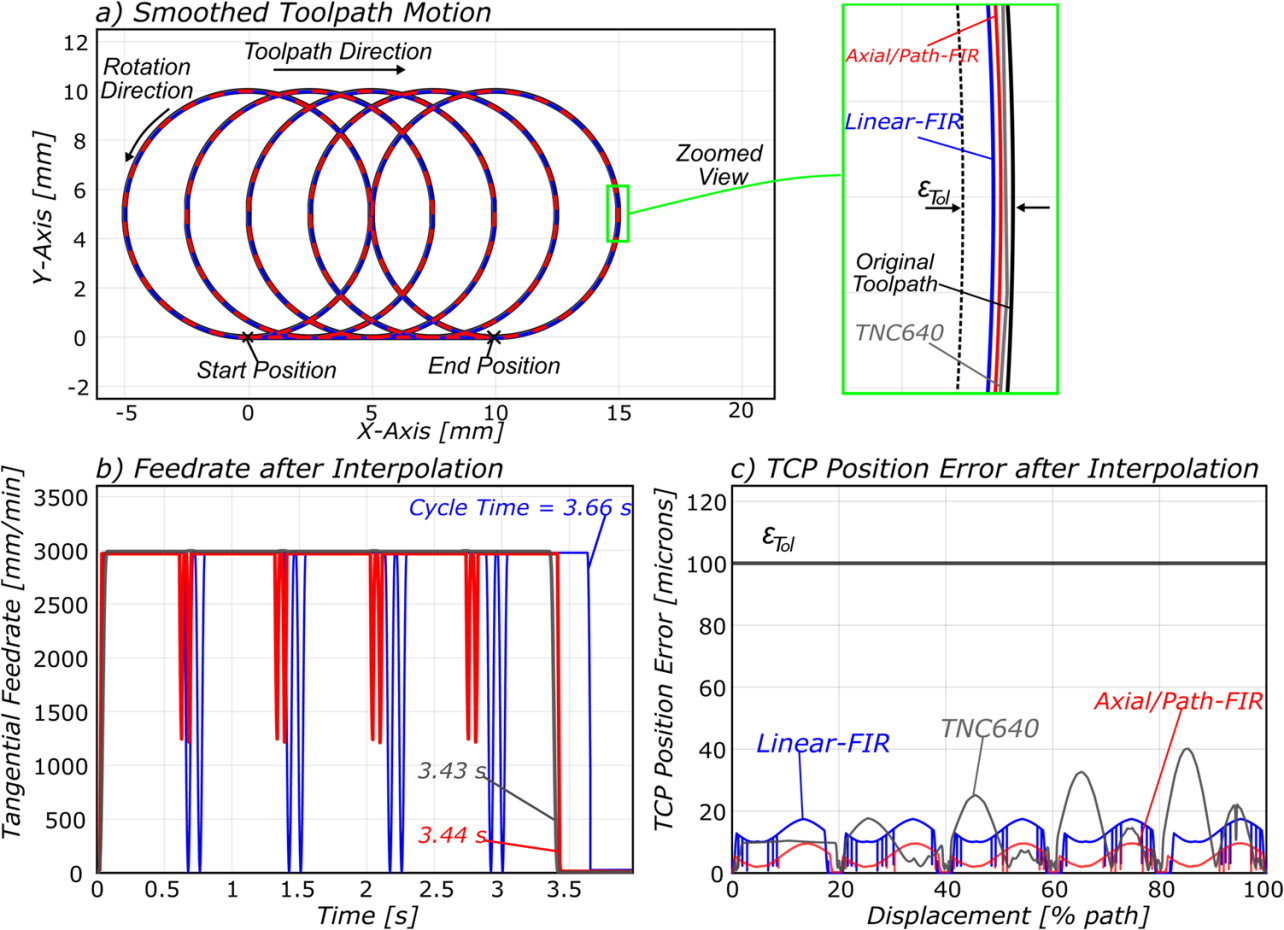
Case study #1 results with **a** the circular trochoidal path used, with radius $R = 5$ mm, feedrate $F = 3000$ mm/min, and TCP tolerance $\varepsilon _{Tol} = 100$ $\mu$m, and **b** the resulting feedrate after interpolation for each interpolator with **c** the resultant TCP position error after interpolation

### Case study #1: G02/G03 Trochoidal Toolpath

The first case study benchmarks the proposed hybrid axial-level/path-level FIR interpolation method. This study involved a circular trochoidal motion toolpath with different feedrates, radii, and TCP tolerances, as seen in Table 2. The proposed axial/path-level FIR is compared against both the linear-FIR from [14] as well as a commercially available NC interpolator, being the Heidenhain TNC640 [31]. The interpolator parameters in Table 1 have been retrieved from the TNC640 archive file and were used across all tests to provide a fair comparison of the interpolator performance. Machine tool operators may configure the CYCLE32 cycle definition [32] to establish the TCP tolerance settings and enable high-speed cutting (HSC) mode. The CYCLE32 command in this trial has been configured for 100-$\mu$m tolerance and with HSC mode enabled. The NC program header with the specified interpolation parameters may be found at Listing 1.

The path was designed in continuous motion and utilised numerical methods to calculate the optimal overlap time $T_{k}$ between G01 and G02/G03 motion commands to ensure blended motion was error-constrained and limited in maximum acceleration and jerk. It must be noted that the overlap time calculation in [14] does not hold in the presence of jerk and acceleration constraints, for it has been proven that acceleration and jerk during the transient circular motion are not monotonically increasing/decreasing (see Eqs. 17, 46, and 50), and thus, overlapping G01 and G02 commands may lead to exceeding jerk constraints—despite satisfying TCP tolerance requirements.

The resulting kinematics are presented in Fig. 9 a and b, and the key performance indicators are compared in Table 2.

**Table 2:** Results for case study #1

				TNC640		Linear-FIR		Axial/path-FIR	
#	Feedrate (mm/min)	Radius (mm)	TCP Tolerance (${\mu }$m)	Limits breached	Cycle time (s)	Limits breached	Cycle time (s)	Limits breached	Cycle time (s)
1	3000	5	10	−	3.43	−	4.14	−	3.44
2	3000	5	100	−	3.43		3.66	−	3.44
3	3000	10	10	−	6.77		7.00	−	6.78
4	3000	10	100	−	6.77	−	7.00	−	6.78
5	6000	5	10	−	3.22	−	7.12	−	1.99
6	6000	5	100	−	1.94	−	2.90	−	1.99
7	6000	10	10	−	4.64	−	9.83	−	3.57
8	6000	10	100	−	3.68	−	3.98	−	3.57

Across all experiments, the TNC640 and the proposed hybrid axial/path-FIR algorithm met TCP tolerance constraints and did not exceed kinematic limits (as defined in Table 1), whereas the linear-FIR algorithm exceeded jerk limits in two of the experiments. This is as expected, for the G02 interpolation strategy used in the linear-FIR algorithm focuses on constraining TCP error, rather than constraining maximum acceleration and jerk around the circular motion. This is further evidenced in the fifth and seventh results, which show that under stricter TCP tolerance constraints, the linear-FIR algorithm has drastically worse performance, resulting in a maximum of 258% greater cycle time compared to the proposed axial/path-FIR in the 6000 mm/min, 5 mm, and $10\, \mu$m experiment. This is due to the linear-FIR algorithm using a feedrate reduction factor to satisfy TCP constraints. The proposed hybrid axial/path-level FIR method outperformed the commercial controller and the linear-FIR algorithm in six of the eight conducted experiments and at worst caused a cycle time elongation of 0.29%. The greatest reduction in cycle time through using the hybrid axial/path-level FIR interpolator can be seen in experiment #5, showing a cycle time reduction of 38.2% compared to that of the TNC640 controller.

The results also show that the proposed hybrid axial/path-FIR outperformed both the commercial and the baseline linear-FIR interpolator in the higher feedrate trials, and most notably in the high-feedrate, low-radius cases, being the tests where feedrate was 6000 mm/min and circle radius was 5 mm. This test has the largest *circular frequency* (with circular frequency defined as $\omega _c = F/R$). The proposed hybrid axial/path-level FIR method is therefore better suited for the smoothing of high-speed, low-radius circular toolpaths, akin to toolpaths used in dynamic milling.

In summary, this case study has evidenced the efficacy of the proposed hybrid Cartesian axial/path-level FIR interpolation algorithm for the interpolation of circular trochoidal toolpaths, showing consistent improvements in performance across experiments. The other notable finding from this case study is the success of programming trochoidal toolpaths using G02 circular arc motions, as all trial toolpaths interpolated with both the industrial interpolator and the FIR interpolation algorithms did not exceed defined TCP tolerance constraints. Furthermore, one can clearly see in Fig. 9b that the linear and axial/path-FIR methods have a very large reduction in feedrate during the transition between linear and circular motions, especially when compared to the TNC640 performance. This is due to the lack of a NC interpolator lookahead function within the proposed algorithm, akin to methods seen in [20, 33]. Should such methods be incorporated in future work, then one may observe further reductions in cycle time for smoothed circular toolpaths.

**Fig. 10:**
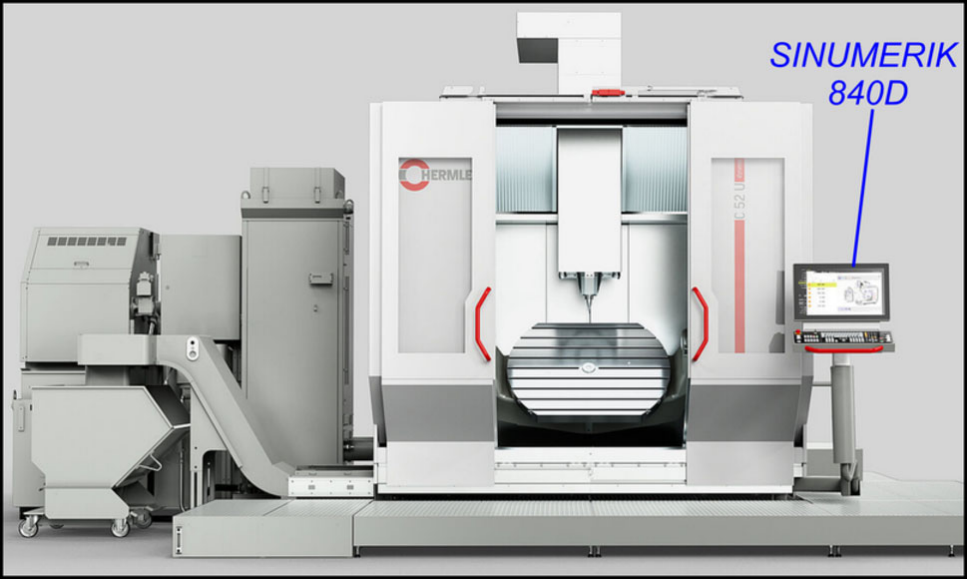
Hermle C52 machining centre [34] with 840D SL controller [35]

**Fig. 11:**
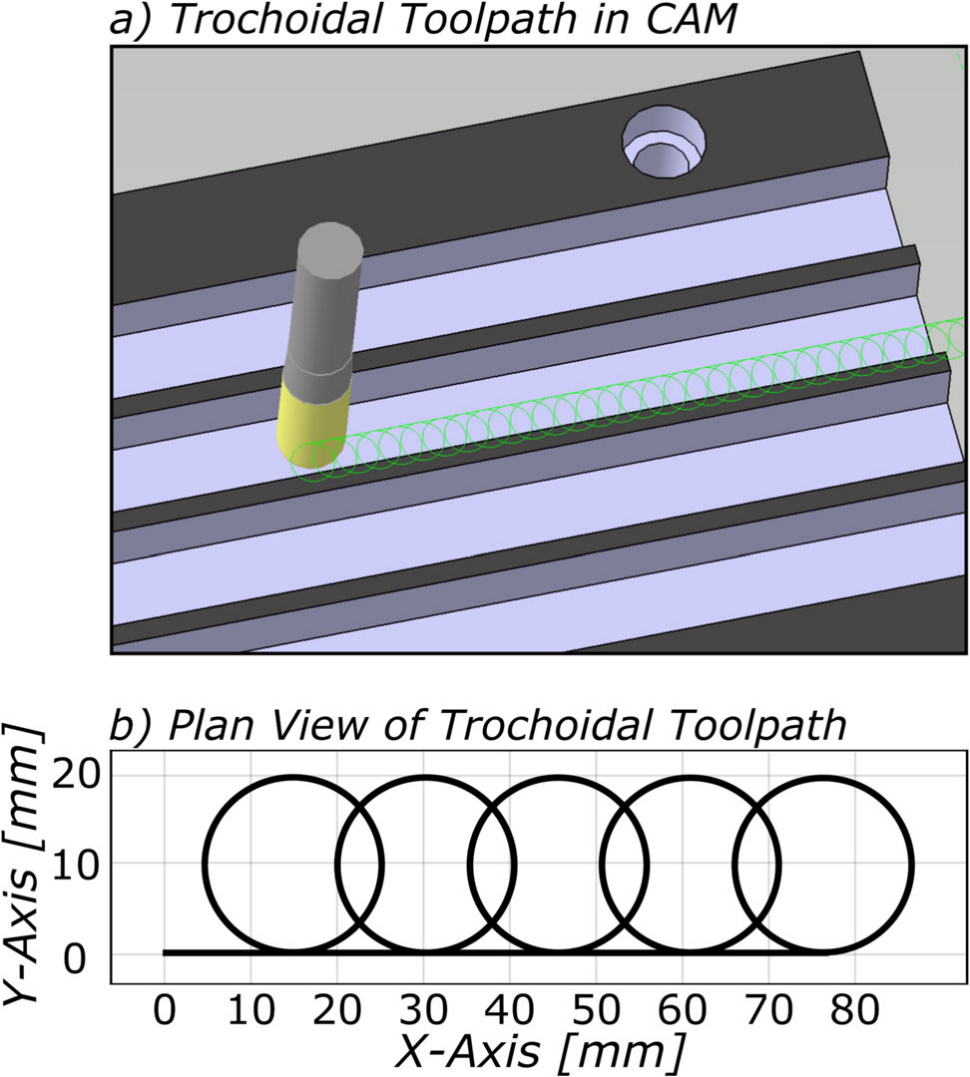
Trochoidal toolpath consisting of circular and linear motion segments displayed **a** in CATIA and **b** in plan view of the truncated path used for case study #2

### Case study #2: G01 vs. G02/G03 trochoidal toolpath

The second case study validates the proposed hybrid axial/path-level FIR method against two commercial NC interpolators: the Siemens 840D controller, and the Heidenhain TNC640. This case study also compared the programming of circular trochoidal motion using G01 commands vs. G02/G03 circular arc motion (Fig. 10).

The 840D experimental trials were conducted on a Hermle C52 5-axis machining centre [34] with a Siemens SINUMERIK 840D SL controller [35]. Machine tool operators have limited control over the interpolation strategy used within the NC kernel, but can call a CYCLE832 command [36] to prioritise machining speed over accuracy, and vice versa. The CYCLE832 command in this trial has been configured for maximum speed and may be found at Listing 2. The TNC640 trials were performed on a Virtual NC using the Heidenhain TNC640 programming station with tolerances configured through the CYCLE32 special cycle (with HSC mode set to 1). Further details on the CYCLE32 configuration may be found at Listing 3. The Hermle C52 was fitted with a SINUMERIK Edge device for high-speed monitoring to record the TCP position signals at 500 Hz, with an excerpt of the machine specifications defined in Table 3.

The baseline toolpath is shown in Fig. 11. This trochoidal toolpath was coded in linear motion form for the 840D tests and in linear/circular output form for the TNC640, after which the novel circular FIR interpolation method was validated on a linear/circular motion trochoidal toolpath.

**Fig. 12:**
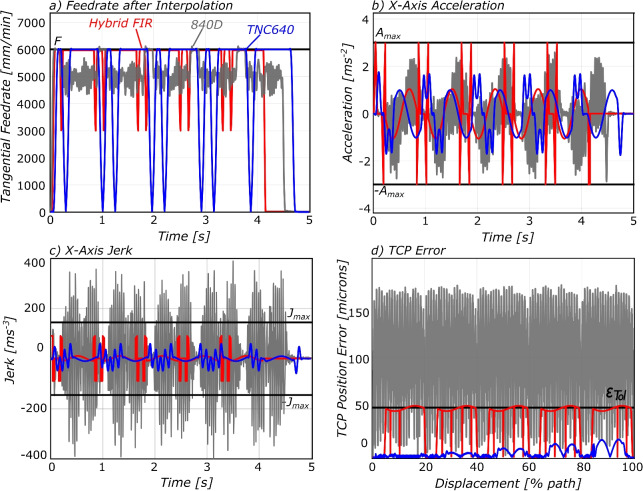
Case study #2 results for trochoidal path with radius *R* = 10 mm, feedrate *F* = 6000 mm/min, and TCP tolerance $\varepsilon _{Tol} = 50$ $\mu$m

The resulting kinematics from the highlighted section in Fig. 11b are presented in Fig. 12 a and d, and the key performance indicators are compared in Table 4.

**Table 3:** Interpolator parameters for case study #2

Controller	Sampling period (ms)	Max. axial acceleration (ms$^{-2}$)	Max. Axial jerk (ms$^{-3}$)
840D	2	3	145
TNC640	3	3	50

**Table 4:** Results for case study #2

				840D		TNC640		Hybrid FIR	
#	Feedrate (mm/min)	Radius (mm)	TCP tolerance (${\mu }$m)	Limits breached	Cycle time (s)	Limits breached	Cycle time (s)	Limits breached	Cycle time (s)
1	3000	10	10		7.82	−	8.58	−	7.90
2	3000	10	100		7.95	−	8.43	−	7.90
3	6000	10	50		4.62		4.89	−	4.09
4	6000	10	100		4.33		4.84	−	4.09

The trends in the first case study are matched in the results of case study 2. The proposed hybrid axial/path-level FIR algorithm outperformed both the 840D and the TNC640 algorithm in the high circular frequency tests (6000 mm/min, 10 mm tests), showcasing *at minimum* a 5.54% reduction in cycle time whilst ensuring interpolated toolpath motion was constrained in both TCP error and acceleration and jerk.

The G01-interpolated trochoidal toolpath (performed using the 840D interpolator) resulted in a breaching of TCP tolerance, whilst the G01-G02/G03 toolpath interpolated with the TNC640 exceeded jerk limits, but *only* in the high circular frequency tests. Utilising circular G02/G03 commands with the proposed axial/path-level FIR algorithms resulted in faster cycle times across three out of four trials, with the assurance that the interpolated toolpaths were constrained in both TCP error and kinematics. The proposed hybrid axial/path-level FIR algorithm showed the greatest performance improvement in experiment #3, with a cycle time reduction of 11.5% against the 840D interpolated path, and a reduction of 16.36% compared to the TNC640 nominal path. These results exemplify the implementation issues of using short-segmented G01 motion for circular trochoidal toolpaths.

### Validation summary

The two case studies demonstrated that the proposed hybrid Cartesian axial/path-level FIR algorithm generates faster and more accurate circular toolpath motion, especially in the presence of strict axial kinematic limits. Improvements in performance are most notable when circular frequency is high, i.e., when feedrate is high and circle radius is small, which is preferential in generating high-speed trochoidal milling toolpaths. These experiments also highlight the implementation issues with programming trochoidal milling using linear (G01) motion. To circumvent the issues caused by the interpolation of highly discretised trochoidal motion, manufacturing engineers should aim to program circular trochoidal motion using circular arc (G02/G03) commands where possible, if constraining both TCP tolerance *and* axial jerk is a priority.

## Conclusions

This paper makes important contributions to the field of numerical control interpolation. The contributions can be summarised as follows:

1. The limitations of FIR interpolation for generating circular toolpaths have been addressed analytically. A novel method of globally satisfying kinematic constraints and TCP position errors during G02/G03 circular motions is presented.
2. The conditions in which FIR interpolation cannot be utilised for Cartesian axial-level smoothing in the presence of kinematic and user-defined error tolerance constraints have been established. These have been overcome by introducing a novel path-level FIR interpolation method that smooths the toolpath in the feed direction with zero interpolation error.
3. A hybrid Cartesian axial/path-level FIR interpolation algorithm has been presented, which optimises the toolpath interpolation strategy based on the fundamental geometry of commanded circular motion.
4. The proposed hybrid axial/path-level FIR algorithm was successfully benchmarked against a commercial CNC controller, showing improved performance during interpolation of a circular trochoidal toolpath composed of G02 commands, with a maximum cycle time reduction of 38.2%.
5. The hybrid axial/path-level FIR interpolation algorithm was validated against a high-performance 5-axis machine tool, demonstrating G02-programmed circular trochoidal motion is superior to G01-coded trochoidal motion with respect to TCP tolerance constraint satisfaction, with a maximum cycle time reduction of 11.5%.

The analyses completed as part of this research focused on circular and trochoidal motion where both the feedrate, circular frequency $\omega _c = F/R$, where the time constant remained constant throughout the toolpath. Further work will expand the analytical solutions to handle toolpaths with non-constant filter parameters and to allow the embedding of the solutions within mixed-feature toolpaths that consist of both linear (G01) and circular (G02/G03) motions.

## Data Availability

Data are available from David Wilkinson with the permission of the University of Sheffield Advanced Manufacturing Research Centre. The data that support the findings of this study are available from the corresponding author, D. Wilkinson, upon reasonable request.

## Appendix A: Cartesian axial-level FIR circular motion analysis

A1 $$\begin{aligned} {s_x}^\prime (t)&= \left\{ \begin{array}{ll} \begin{aligned} &  \frac{R}{\omega _c T_1}\Big ( \sin \left( \omega _c t +\theta _s \right) - \omega _c t \cos \left( \theta _s\right) - \sin \left( \theta _s\right) \Big ) \\ &  + x_c + R\cos \left( \theta _s\right) \end{aligned} &  0 \le t< T_1, \\ \frac{2R}{{\omega _c} T_1}\sin \left( \frac{\omega _c T_1}{2}\right) \sin \left( \omega _c t + \phi \right) + x_c &  T_1 \le t< T_v, \\ \begin{aligned} &  \frac{R}{T_1}\big ( t - T_v\big )\cos \left( \omega _c T_v + \theta _s\right) \\   &  - \frac{R}{\omega _c T_1}\sin \left( \omega _c t -\omega _c T_1 + \theta _s\right) \\   & + \frac{R}{\omega _c T_1}\sin \left( \omega _c T_v - \omega _c T_1 + \theta _s\right) \\   & + \frac{2R}{{\omega _c} T_1}\sin \left( \frac{\omega _c T_1}{2}\right) \sin \left( \omega _c T_v + \phi \right) + x_c \end{aligned}&  T_v \le t < T_v + T_1, \\ R\cos \left( \theta _e\right) + x_c &  T_v + T_1 \le t. \end{array} \right. \end{aligned}$$

A2 $$\begin{aligned} {s_y}^\prime (t)&= \left\{ \begin{array}{ll} \begin{aligned} &  -\frac{R}{\omega _c T_1}\Big ( \cos \left( \omega _c t +\theta _s \right) -\cos \left( \theta _s \right) + \omega _c t \sin \left( \theta \right) \Big ) \\ &  + y_c + R\sin \left( \theta _s\right) \end{aligned} &  0 \le t< T_1, \\ -\frac{2R}{{\omega _c} T_1}\sin \left( \frac{\omega _c T_1}{2}\right) \cos \left( \omega _c t + \phi \right) + y_c &  T_1 \le t< T_v, \\ \begin{aligned} &  \frac{R}{T_1}\big ( t - T_v\big )\sin \left( \omega _c T_v + \theta _s\right) \\   & + \frac{R}{\omega _c T_1}\cos \left( \omega _c t -\omega _c T_1 + \theta _s\right) \\   &  - \frac{R}{\omega _c T_1}\cos \left( \omega _c T_v - \omega _c T_1 + \theta _s\right) \\ & -\frac{2R}{{\omega _c} T_1}\sin \left( \frac{\omega _c T_1}{2}\right) \cos \left( \omega _c T_v + \phi \right) + y_c \end{aligned}&  T_v \le t < T_v + T_1, \\ R\sin \left( \theta _e\right) + y_c&  T_v + T_1 \le t. \end{array} \right. \end{aligned}$$

## Appendix B: Path-level FIR interpolated Cartesian axial motion profiles - *y*-axis

B3 $$\begin{aligned} {s_y}^\prime (t)&= \left\{ \begin{array}{ll} y_c + R\sin \left( \frac{\omega _c}{2T_1} t^2+ \theta _s \right) &  0 \le t< T_1, \\ y_c + R\sin \left( \omega _c t - \frac{\omega _c T_1}{2}+ \theta _s \right) &  T_1 \le t< T_v \\ y_c + R\sin \left( -\frac{\omega _c}{2T_1} \tau (t)^2 - \omega _c T_v + \theta _s \right) &  T_v \le t < T_v + T_1 \\ y_c + R\sin \left( \omega _c T_v+ \theta _s \right) &  T_v + T_1 \le t. \end{array} \right. \end{aligned}$$

B4 $$\begin{aligned} {v_y}^\prime (t)&= \left\{ \begin{array}{ll} \frac{\omega _c R}{T_1} t \cos \left( \frac{\omega _c}{2T_1} t^2+ \theta _s \right) &  0 \le t< T_1, \\ \omega _c R \cos \left( \omega _c t - \frac{\omega _c T_1}{2}+ \theta _s \right) &  T_1 \le t< T_v \\ -\frac{\omega _c R}{T_1} \tau (t) \cos \left( -\frac{\omega _c}{2T_1} \tau (t)^2 - \omega _c T_v + \theta _s \right) &  T_v \le t < T_v + T_1 \\ 0 &  T_v + T_1 \le t. \end{array} \right. \end{aligned}$$

B5 $$\begin{aligned} {A_y}^\prime (t)&= \left\{ \begin{array}{ll} \begin{aligned} &  \frac{\omega _c R}{T_1} \cos \left( {\theta _1}^\prime (t)+ \theta _s \right) \\ &  - \frac{{\omega _c}^2 R}{{T_1}^2}t^2 \sin \left( {\theta _1}^\prime (t)+ \theta _s \right) \end{aligned} &  0 \le t< T_1, \\ - {\omega _c}^2 R\sin \left( {\theta _2}^\prime (t)+ \theta _s \right) &  T_1 \le t< T_v, \\ \begin{aligned} &  - \frac{ \omega _c R}{T_1} \cos \left( {\theta _3}^\prime (t)+ \theta _s \right) \\ &  - \frac{ {\omega _c}^2 R}{{T_1}^2}\tau (t) ^2 \sin \left( {\theta _3}^\prime (t)+ \theta _s \right) \end{aligned} &  T_v \le t < T_v + T_1 , \\ 0 &  T_v + T_1 \le t. \end{array} \right. \end{aligned}$$

B6 $$\begin{aligned} {J_y}^\prime (t)&= \left\{ \begin{array}{ll} \begin{aligned} &  - \frac{3 {\omega _c}^2 R}{{T_1}^2}t \sin \left( {\theta _1}^\prime (t)+ \theta _s \right) \\ &  -\frac{{\omega _c}^3 R}{{T_1}^3}t^3 \cos \left( {\theta _1}^\prime (t)+ \theta _s \right) \end{aligned} &  0 \le t< T_1, \\ -{\omega _c}^3 R\cos \left( {\theta _2}^\prime (t)+ \theta _s \right) &  T_1 \le t< T_v, \\ \begin{aligned} &  - \frac{3 {\omega _c}^2 R}{{T_1}^2}\tau (t) \sin \left( {\theta _3}^\prime (t) + \theta _s \right) \\ &  + \frac{{\omega _c}^3 R}{{T_1}^3}\tau (t) ^3 \cos \left( {\theta _3}^\prime (t) + \theta _s \right) \end{aligned} &  T_v \le t < T_v + T_1 , \\ 0 &  T_v + T_1 \le t. \end{array} \right. \end{aligned}$$
